# Virtual Trauma-Focused Therapy for Military Members, Veterans, and Public Safety Personnel With Posttraumatic Stress Injury: Systematic Scoping Review

**DOI:** 10.2196/22079

**Published:** 2020-09-21

**Authors:** Chelsea Jones, Antonio Miguel-Cruz, Lorraine Smith-MacDonald, Emily Cruikshank, Delaram Baghoori, Avneet Kaur Chohan, Alexa Laidlaw, Allison White, Bo Cao, Vincent Agyapong, Lisa Burback, Olga Winkler, Phillip R Sevigny, Liz Dennett, Martin Ferguson-Pell, Andrew Greenshaw, Suzette Brémault-Phillips

**Affiliations:** 1 Heroes in Mind Advocacy and Research Consortium Faculty of Rehabilitation Medicine University of Alberta Edmonton, AB Canada; 2 1 Field Ambulance Physical Rehabilitation Department Canadian Armed Forces Health Services Department of National Defense Edmonton, AB Canada; 3 Department of Occupational Therapy Faculty of Rehabilitation Medicine University of Alberta Edmonton, AB Canada; 4 Glenrose Rehabilitation Hospital Research Innovation and Technology (GRRIT) Glenrose Rehabilitation Hospital Edmonton, AB Canada; 5 Department of Educational Psychology Faculty of Education University of Alberta Edmonton, AB Canada; 6 Rehabilitation Science Faculty of Rehabilitation Medicine University of Alberta Edmonton, AB Canada; 7 Department of Psychiatry Faculty of Medicine and Dentistry University of Alberta Edmonton, AB Canada; 8 Scott Health Sciences Library University of Alberta Edmonton, AB Canada

**Keywords:** trauma, mental health, telemedicine, therapy, rehabilitation, digital health, psychotherapy, military, veteran, first responder, public safety personnel, teletherapy, psychotherapy, telepsychiatry, mobile phone

## Abstract

**Background:**

A necessary shift from in-person to remote delivery of psychotherapy (eg, teletherapy, eHealth, videoconferencing) has occurred because of the COVID-19 pandemic. A corollary benefit is a potential fit in terms of the need for equitable and timely access to mental health services in remote and rural locations. Owing to COVID-19, there may be an increase in the demand for timely, virtual delivery of services among trauma-affected populations, including public safety personnel (PSP; eg, paramedics, police, fire, correctional officers), military members, and veterans. There is a lack of evidence on the question of whether digital delivery of trauma-therapies for military members, veterans, and PSP leads to similar outcomes to in-person delivery. Information on barriers and facilitators and recommendations regarding digital-delivery is also scarce.

**Objective:**

This study aims to evaluate the scope and quality of peer-reviewed literature on psychotherapeutic digital health interventions delivered remotely to military members, veterans, and PSP and synthesize the knowledge of needs, gaps, barriers to, and facilitators for virtual assessment of and virtual interventions for posttraumatic stress injury.

**Methods:**

Relevant studies were identified using MEDLINE (Medical Literature Analysis and Retrieval System Online), EMBASE (Excerpta Medica dataBASE), APA (American Psychological Association) PsycINFO, CINAHL (Cumulative Index of Nursing and Allied Health Literature) Plus with Full Text, and Military & Government Collection. For collation, analysis, summarizing, and reporting of results, we used the CASP (Critical Skills Appraisal Program) qualitative checklist, PEDro (Physiotherapy Evidence Database) scale, level of evidence hierarchy, PRISMA-ScR (Preferred Reporting Items for Systematic Reviews and Meta-Analyses extension for Scoping Reviews), and narrative synthesis.

**Results:**

A total of 38 studies were included in this review. Evidence for the effectiveness of digital delivery of prolonged exposure therapy, cognitive processing therapy, behavioral activation treatment with therapeutic exposure to military members, veterans, and PSP was rated level 1a, whereas evidence for cognitive behavioral therapy was conflicting. The narrative synthesis indicated that virtual delivery of these therapies can be as effective as in-person delivery but may reduce stigma and cost while increasing access to therapy. Issues of risk, safety, potential harm (ie, suicidality, enabling avoidance), privacy, security, and the match among the therapist, modality, and patient warrant further consideration. There is a lack of studies on the influences of gender, racial, and cultural factors that may result in differential outcomes, preferences, and/or needs. An investigation into other therapies that may be suitable for digital delivery is needed.

**Conclusions:**

Digital delivery of trauma therapies for military members, veterans, and PSP is a critical area for further research. Although promising evidence exists regarding the effectiveness of digital health within these populations, many questions remain, and a cautious approach to more widespread implementation is warranted.

## Introduction

### Background

Physical distancing during the COVID-19 pandemic has led to a rapid paradigm shift toward remote mental health service delivery and a surge in the use of digital health delivery (eg, teletherapy, telemedicine, eHealth, and mobile health) [1,2]. With the onset of COVID-19 and physical distancing rules for the containment of infection spread, supporting people at a physical distance with digital health delivery methods became necessary for accessing services, screening, assessment, and treatment [2,3]. Many aspects of legal, clinical, cultural, practical, and privacy and/or security issues remain to be addressed for delivering mental health services to trauma-affected populations, including public safety personnel (PSP; eg, border services, communications officials, correctional workers, firefighters, paramedics, police, etc), military members, and veterans [4]. mental health concerns and posttraumatic stress injuries (PTSIs) in these groups may be associated with professional service during, or exacerbated by, the COVID-19 pandemic. This review aims to systematically identify the scope of what is known in this context and to summarize current evidence supporting the use of digital health with military members, veterans, and PSP, together with a discussion of barriers and facilitators.

Military members and PSP are frequently exposed to potentially high stress and traumatic experiences in the course of service [5]. Such exposures can impact their mental and psychosocial health and result in PTSIs, a range of challenges from posttraumatic stress disorder (PTSD) to symptom clusters that may not meet diagnostic criteria but interfere with daily functioning in social, work, or family activities [4]. PTSD is the most common PTSI experienced by military members and veterans [6-8] and remains to be the predominant focus of most military and veteran health research and care [9-11]. Characterized by intrusion symptoms, avoidance, changes in cognition and mood, and changes in arousal and reactivity [12], PTSD has historically been difficult to treat because of the variety of associated symptoms. Isolated or cumulative traumatic experiences can also cause long-term psychological and spiritual struggles, including depression, anxiety, and moral injury [9,11,13,14]. Moral injury, a separate trauma syndrome that results from exposure to morally injurious experiences such as witnessing or participating in acts that transgress personal morals and values [14], is potentially a key PTSD comorbidity [15].

The incidence of PTSIs among military members, veterans, and PSP varies: within military and veteran populations globally, PTSD prevalence is persistent, complex, and may increase over time [16,17]. Among US military members deployed during the War on Terror, PTSD prevalence estimates reached 19% [17] compared with 5.3% for Canadians [18], 2.7% to 4% in UK military members [19], and 3% for military members from the Netherlands [20]. In 2010, the rate of PTSD among the Australian Defense Force was reported to be 8.3% [21]. A recent meta-analysis reported that overall rates remained high (approximately 23%) for post–9/11 US veterans [22], and PTSD increased to 16% for Canadian veterans [23]. Global PTSI studies among PSP also demonstrate elevated prevalence, severity, and complexity. Prevalence of PTSIs among PSP in a recent Canadian study was reported as follows: municipal police, 36.7%; firefighters, 34.1%; Royal Canadian Mounted Police, 50.2%; and paramedical staff, 49.1% [24].

Multiple gold-standard frontline psychotherapeutic interventions have been used to treat PTSIs in these populations, including prolonged exposure therapy (PE), cognitive processing therapy (CPT), cognitive behavioral therapy (CBT), and eye movement desensitization reprocessing (EMDR). PE and CPT have been the main research focus over the last 25 years and appear to have the greatest efficacy for PTSD [25-27]. Although mental health interventions have been predominantly delivered in-person, the pandemic and geographical barriers impede in-person access to assessment and interventions. On the basis of recent evidence-based publications, some of the main barriers to veterans and military members seeking treatment include concerns about the specific treatment itself (ie, CBT, PE, CPT), mental health stigma from self and others, and technological logistics, including internet quality, familiarity with software, and competence with technology [28]. Furthermore, reduced access to mental health clinicians who are adequately trained in the remote delivery of therapy and individual factors, such as unfavorable attitudes toward interventions, may also be significant barriers to receiving and benefiting from digital health treatments [29]. There is very limited access to specialized mental health services in active theaters of military operations, in remote locations, and for those living in rural communities [30]. The new reality of COVID-19 physical distancing requirements adds to existing barriers for those who may require medical treatment and services for mental and physical health. Increasing access to and understanding the effectiveness and limitations of virtual care is now essential.

Digital health (eg, teletherapy, telemedicine, eHealth, and mobile health) may offer military members, veterans, and PSP alternative access to mental health services and therapies in a timely manner. Current literature identifies accepted conventional benefits associated with in-person delivery of gold-standard frontline psychotherapies, including flexible delivery times, physical privacy that enables avoidance of stigma, enhanced self-efficacy, and minimized negative attitudes toward mental health interventions [29,31]. In contrast, potential concerns about the provision of therapy via digital health include potential practical issues with technology interactions, client willingness to engage in telehealth, privacy, and safety concerns (eg, how adverse events will be handled remotely), and mental health clinician attitudes.

Although there is evidence regarding the general use of digital health, significant knowledge gaps exist regarding its use in this context, including an understanding of needs, barriers, and facilitators of the use, uptake, and sustainability of digital health; technological issues that may impede the use of digital health solutions (eg, feasibility, logistics, security, firewalls, compatibility, and policies in military and PSP organizations) and technology acceptance by PSP, military members, veterans, and mental health clinicians; and evidence of clinical effectiveness of remote digital assessment and treatment of PTSIs. There is also, as yet, a general lack of published work focusing on knowledge dissemination and implementation plans that will ensure that timely, accessible, and relevant evidence is available to decision makers who may consider the adoption of virtual delivery of assessment and interventions. This review explores the literature regarding digitally delivered psychotherapeutic interventions for military members, veterans, and PSP, along with barriers, facilitators, and recommendations for its use. This study contributes uniquely to mental health response to COVID-19 by summarizing the evidence for realistic digital health solutions for the delivery of mental health services for military members, veterans, and PSP with PTSIs for whom mental health challenges may be associated with or exacerbated by the COVID-19 pandemic.

The research questions guiding this systematic scoping review are as follows:

1. What is the quality of the existing literature addressing digital health delivery of gold-standard psychotherapeutic trauma interventions for military members, veterans, and PSP with trauma-related mental illness?
2. What evidence exists on the efficacy of digital health delivery of gold-standard psychotherapeutic trauma interventions for military members, veterans, and PSP with trauma-related mental illness compared with regular in-person intervention delivery?
3. What are the facilitators, barriers, themes, clinical recommendations, considerations, and knowledge gaps in the current peer-reviewed, evidence-based literature regarding digital health delivery of PTSI-trauma interventions for military members, veterans, and PSP with trauma-related mental illness?

### Objectives

This scoping review aims to (1) systematically evaluate the quality of the existing quantitative, qualitative, and mixed methods peer-reviewed literature on digital health interventions for mental health with military member, veteran, and PSP populations and (2) synthesize the knowledge of needs, gaps, barriers, and facilitators for digital health delivery of PTSI assessment and interventions based on the existing literature.

## Methods

### Identification of Relevant Studies

This scoping review employed the following overarching steps: (1) formulation of the research questions based on PICO (Population, Intervention, Comparison, and Outcome) guidelines, (2) identification of relevant studies, (3) selection of studies, (4) charting of the data, and (5) collation, analysis, summarization, and reporting of results [32]. This scoping review follows the PRISMA-ScR (Preferred Reporting Items for Systematic Reviews and Meta-Analyses extension for Scoping Reviews) reporting guidelines [33].

#### Information Sources and Search Strategy

The research team developed a search strategy based on specific inclusion and exclusion criteria. It included the following databases: MEDLINE (Medical Literature Analysis and Retrieval System Online; Ovid MEDLINE ALL), EMBASE (Excerpta Medica dataBASE; Ovid interface), APA (American Psychological Association) PsycINFO (Ovid interface), CINAHL (Cumulative Index of Nursing and Allied Health Literature) Plus with Full Text (EBSCOhost interface), and Military & Government Collection (EBSCOhost interface). The search consisted of an extensive list of keywords and subject headings covering 4 concepts: (1) traumatized individuals, (2) military or rescue personnel, (3) person therapy conducted remotely through technology (telephone, videoconferencing, or online), and (4) specific trauma-informed therapies (Multimedia Appendix 1). The 4 concepts were then combined with the Boolean AND. The search was limited to articles published from 2010 onward to include only current technology and therapeutic techniques. Studies were also limited to peer-reviewed articles in the English language. Editorials and other nonresearch articles were removed where possible. The full search strategy is available in Multimedia Appendix 1.

#### Inclusion and Exclusion Criteria

Articles selected for inclusion in this scoping review addressed military members, veterans, and/or PSP who had a primary diagnosis of PTSD and/or a trauma-related mental health disorder. The intervention modality in outcome studies, or being targeted for validity or reliability studies or reviews, was limited to psychotherapeutic treatments administered via a remote platform. Psychotherapeutic interventions most strongly recommended for military members, veterans, and PSP include CPT, CBT, PE, and EMDR [34-38]. Other trauma-informed therapies were also included in the search, including motivational interviewing, adaptive disclosure therapy (ADT), and accelerated resolution therapy (ART), although the search terms would include results containing other trauma therapies [39,40]. Remote psychotherapeutic interventions were limited to those delivered via a remote person-to-person interaction (ie, the participant and clinician were in separate physical locations during intervention delivery). The delivery platforms included videoconferencing, text, app-based, virtual reality, or telephone communication, provided interaction between the participant and clinician was performed in real time and not prerecorded or automated. Both individual and group interventions were included.

If the published work included participants with comorbid conditions, such as other mental health disorders, disrupted sleep, chronic pain, substance use disorder, or traumatic brain injury, it was included if the additional conditions were secondary to the trauma-induced diagnosis and not the primary focus of the specific research study.

All articles included in the data set were peer reviewed and were quantitative, qualitative, mixed methods, and meta-analyses, regardless of positive, negative, or neutral findings. Articles were excluded from the review if they did not meet the inclusion criteria, were not peer reviewed, or were commentaries, editorials, or gray literature such as nonpublished graduate student theses. Studies that exclusively addressed civilians, such as those that focused on family members of military, veteran, and PSP populations only, were also excluded. Studies of interventions that were not specific to trauma, such as dialectical behavioral therapy, were excluded from the final selection of articles.

### Selection of Studies

The study selection phase followed a variation of the procedures used by Neubauer et al [41] and Miguel Cruz et al [42]. First, a member of the research team exported all of the identified studies to the reference manager software EndNote X9.3.2 (Thomson Reuters) [43]. After deduplication, the references were imported to Covidence [44]. Second, before the title and abstract evaluation phase, members of the research team were trained in applying the inclusion and exclusion criteria (calibration phase). Then, 2 pairs of independent researchers evaluated the titles and abstracts of the remaining studies and compared them with the inclusion and exclusion criteria. Differences between the 2 pairs of independent researchers regarding the decision of whether or not to include a study in the next phase were addressed at subsequent meetings. During the full paper reading phase, 2 researchers reviewed the full texts of the selected studies. Each researcher independently assessed the studies to determine their suitability for inclusion in the data extraction phase. Inclusion or exclusion into the data set for analysis required consensus.

### Charting of the Data: Data Extraction Process

During this phase, the researchers completed the data extraction of the final selected papers and met regularly to reconcile the differences that arose through discussion. In case of any disagreement, one of the researchers acted as a third rater. In addition, the co–principal investigators validated the data extracted from the studies. In each selected study, the research team extracted data according to the following domains: population (medical condition, age, branch of the military, race or ethnicity, sample size [N], and mean age [SD] in years), study features (design, outcome variables, and assessment tools), intervention (whether the intervention was provided via remote access, mode of delivery, therapy or care delivered, or in a group or 1:1 session), clinical assessment, clinical outcome measures, clinical outcomes, assessment of technology usability, technology outcome measures, technology, use outcomes, duration, and data analysis strategies.

### Analysis, Summarization, and Reporting

#### Data Analysis

All data were analyzed and validated by at least two team members involved in the analysis. The research team met regularly to discuss data extraction, analysis, and synthesis, which were iterative and, in some cases, concurrent. Any discrepancies in the analysis of quantitative or qualitative data were resolved through discussion. This nonlinear process guaranteed rigor and internal validity.

A thematic analysis and narrative synthesis were used to qualitatively analyze the studies and compile the results. Data immersion occurred before the commencement of the analysis and coding process. The thematic analysis involved examining the text in detail to identify recurring patterns (*themes*) through both inductive and deductive reasoning [45]. The framework by Braun and Clarke [45] for qualitative thematic analysis guided the inductive analysis such that no pre-existing coding frame was imposed on the studies. The deductive analysis was guided by the research questions, particularly barriers, facilitators, and recommendations associated with the use of digital health [45]. Once the coding structure was developed, the first round of coding was completed by 2 team members. Open codes were later combined into preliminary patterns focusing on similarities and differences within and between studies. More abstract concepts were assigned to broader categories of themes and verified through key quotes. The data were then reviewed and recoded twice. Team members continually compared themes and resolved their differences through discussion. Following the thematic analysis, a narrative synthesis was conducted to organize, describe, explore, interpret, and fundamentally tell the story of the analysis [46,47].

#### Quality of the Evidence

The 38 selected studies were further analyzed to determine the quality of the evidence. The 3 tools utilized for this step were the PEDro (Physiotherapy Evidence Database) scale [48], CASP (Critical Appraisal Skills Program) qualitative checklist [49], and the levels of evidence hierarchy. The levels of evidence used to summarize the findings are based on the levels of evidence developed by Straus et al [50,51]. The CASP qualitative checklist can be utilized as a tool to evaluate whether qualitative literature is valuable to the research topic of interest. The PEDro scale can assist researchers in rapidly identifying which randomized controlled trials (RCTs) are likely to be internally valid and could have sufficient statistical information to make their results interpretable [48]. For RCTs, studies scoring 9 to 10 on the PEDro scale are considered to be of *excellent* methodological quality. Studies with PEDro scores ranging from 6 to 8 are considered to be of *good* quality, whereas studies scoring 4 or 5 were of *fair* quality. Studies that scored below 4 were considered to be of *poor* quality [50].

At least two researchers evaluated each of the included studies with the appropriate tool, with the PEDro scale being applied to RCTs (n=26) and the CASP qualitative checklist being applied to studies with a qualitative component (n=4). Each study with a quantitative component with outcome variables was then categorized based on the trauma therapy intervention used in the study and assigned a grade based on the levels of evidence [50,51].

## Results

### Search Results

**Figure 1:**
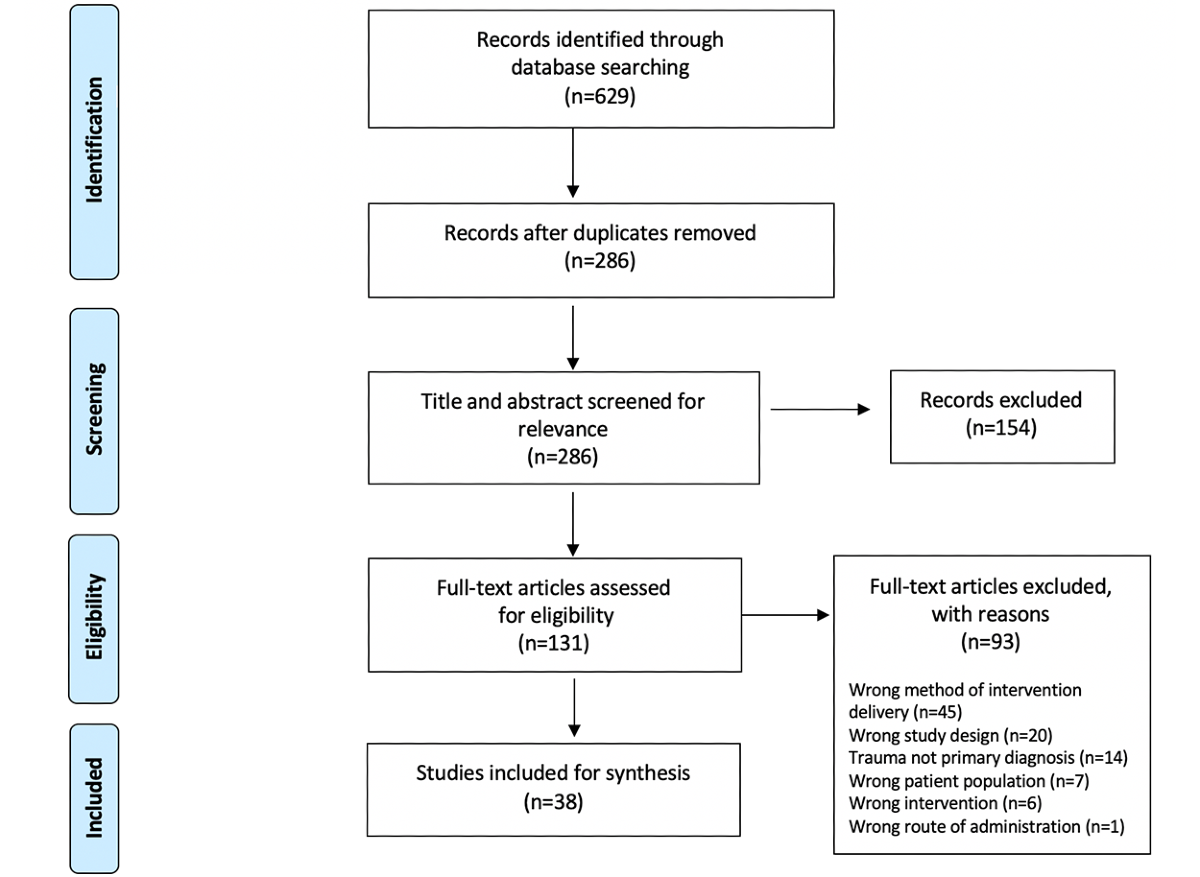
A PRISMA (Preferred Reporting Items for Systematic Reviews and Meta-Analyses) extension for Scoping Reviews chart of the scoping review study identification, selection, exclusion, and inclusion.

The search strategy yielded 629 articles (see the PRISMA diagram in Figure 1). After deduplication, 286 titles and abstracts were screened. A total of 131 full-text documents were reviewed, with 93 being excluded (Figure 1) for reasons of including a therapeutic intervention not specific to traumatic stress-related disorders, not involving military members, veterans, or PSP, not being peer reviewed, the diagnosis not being related to trauma, or the administration of the therapeutic intervention not being via digital health. The remaining 38 studies were included in the review. Results of the descriptive analysis are displayed in Multimedia Appendix 2. In Multimedia Appendix 3 [25,28,52-87], a summary of the results of each included study is displayed.

### Evidence Appraisal Results

The RCTs (n=29) varied in quality, with PEDro scores ranging from 4 to 9. The CASP qualitative scale found all studies with a qualitative component (n=4) to be valuable and to have contributed to the overall evidence on the topic. Levels of evidence (Table 1) ranged from 1a (PE, CPT, and behavioral activation [BA] and therapeutic exposure [BA-TE]) to 4 BA. A level 1a rating indicates strong evidence and is achieved when 2 or more RCTs of high quality (PEDro ≥6) demonstrate similar findings, and a level 4 rating indicates that findings are supported by at least one study of pre- and posttest, posttest, case series, or single-subject intervention design [50]. The results for CBT were conflicting (Table 1).

**Table 1:** Level of evidence hierarchy for digital health delivery of trauma therapy interventions utilized with military members, veterans, and public safety personnel.

Treatment	Conclusion of level of evidence	Study
Prolonged exposure therapy	There is level 1a evidence that prolonged exposure therapy delivered via videoconferencing significantly reduces PTSD^a^ symptoms in veterans and military members with PTSD	Yuen et al, 2015 [25] Wierwille et al, 2016 [53] Tuerk et al, 2010 [58] Morland et al, 2019 [83] Jaconis et al, 2017 [70] Hernandez-Tejada et al, 2017 [72] Gros et al, 2011 [74] Gros et al, 2018 [75] Franklin et al, 2017 [77] Pelton et al, 2015 [80] Olden et al, 2017 [81] Acierno et al, 2017 [86]
Cognitive processing therapy	There is level 1a evidence that cognitive processing therapy delivered via videoconferencing significantly reduces PTSD symptoms in veterans with PTSD	Wierwille et al, 2016 [53] Wells et al, 2019 [56] Morland et al, 2015 [63] Morland et al, 2014 [64] Morland et al, 2011 [66] Maieritsch et al, 2016 [67] Fortney et al, 2015 [78] Murphy and Turgoose, 2019 [82]
Cognitive behavioral therapy	There is conflicting evidence that cognitive behavioral therapy delivered via videoconferencing or telephone significantly reduces PTSD symptoms in veterans and military members with PTSD	Ziemba et al, 2014 [52] Stecker et al, 2014 [28] Stecker et al, 2016 [62] Gallegos et al, 2016 [76] Trahan et al, 2016 [59]
Behavioral activation and therapeutic exposure	There is level 1a evidence that behavioral activation and therapeutic exposure delivered via home-based videoconferencing significantly reduces PTSD symptoms in veterans with PTSD	Acierno et al, 2016 [87] Strachan et al, 2012 [60]
Behavioral activation treatment for PTSD	There is level 4 evidence that behavioral activation for PTSD delivered via clinic-based videoconferencing significantly reduces PTSD symptoms and depressive symptoms in military members with PTSD	Luxton et al, 2015 [68]

^a^PTSD: posttraumatic stress disorder.

### Qualitative Analysis Results

The narrative synthesis and qualitative thematic analysis yielded a number of emerging themes related to the efficacy, clinical utility, ethics, accessibility, facilitators and barriers regarding the remote delivery of trauma-focused therapies. In addition, a number of clinical recommendations have emerged that complement the findings of the quantitative analysis and rating of evidence.

#### Facilitators

A number of facilitators were identified regarding the virtual delivery of trauma therapies to military members, veterans, and PSP (Table 2). These included (1) the convenience of accessing teletherapy, particularly for clients in rural and remote areas [79,85,87], (2) the comfort of participating in therapy from the client’s home [57,59,85,87] resulting in less stress [55,58] and stigma, [86,87], (3) the efficaciousness of several different evidence-based PTSD treatment modalities delivered using digital health, including PE, CPT, CBT, and BA-TE [25,53,56,77], and (4) the ability to see a therapist from a central health clinic or the location of their choosing reduced travel time and transportation and missed work costs [25,55,82]. Many clients found that participating in teletherapy provided the same opportunities for relationship building with the therapist as in-person treatment [52,81]. From the mental health clinician perspective, the same clinical skills and safety protocols required for handling increased emotional responses and symptom emergence in-person were noted to be used in a remote delivery context [58].

**Table 2:** Summary of facilitators from all studies included in the review.

Themes and subthemes			Findings	Illustrative quotes
**1. Facilitators**				
	**1.1 Participants**			
		1.1.1 Availability and accessibility of services	Access. Teletherapy may be beneficial for individuals who live in rural areas, as they are more accessible than in-person services. This benefit may be increased if the internet or electronic devices are provided to the clients	“Service members who are living in geographically remote locations or in areas that have a shortage of specialty healthcare professionals may especially benefit from Home-Based Tele Mental Health options.” [88]
		1.1.2 Ethnic background and sex	Ethnicity and sex. Black veterans seem to be more likely to seek out services, whether through telehealth or in-person. Additionally, female military veterans may be more open to using teletherapy compared to in-person treatment	“Blacks overall were found to be more than 2 times as likely to seek treatment as White participants.” [62] “...telehealth may help to overcome unique barriers experienced by female Veterans seeking care in a traditionally male-dominated health care system. Adoption of telehealth technologies may be particularly useful as the VAMC continues its efforts to expand services sensitive to the experiences of female Veterans including an expanded awareness and focus on providing MST related services.” [70]
		1.1.3 Rapport/trust building in therapy	Rapport. The ability to build rapport and develop a strong therapeutic alliance is possible with teletherapy and has been demonstrated in a number of studies	“Participants reported high levels of therapeutic alliance with their therapist throughout the treatment.” [81]
		1.1.4 Participant environment	Home environment. Having the client participate in therapy from their own home allows them to feel comfortable and engage more easily in the therapeutic process. This may be especially helpful for clients who have experienced military sexual trauma	“Participants mentioned that being able to do therapy in their own environment helped them to relax and engage better than if they had had to go somewhere unfamiliar.” [85]
		1.1.5 Use or uptake of the therapy	Uptake. Evidence seems to point to the comfort of clients with the use of teletherapy, belief in its effectiveness, and a willingness to use it again	“...throughout the duration of treatment, the majority of participants reported that they would be willing to use telehealth-delivered treatment again.” [81] “Participants also endorsed high expectations that the intervention would be helpful throughout the course of treatment.” [81]
	**1.2 Technology**			
		1.2.1 Stigma associated with therapy	Stigma. There seems to be a reduced amount of stigma surrounding teletherapy compared to in-person due to issues of privacy	“The Advantage of Home-Based Teletherapy include reduced stigma (eg, patients do not need to visit a mental health care facility)...” [87]
		1.2.2 Availability or accessibility or cost-effectiveness of services	Cost. Home-based teletherapy may be less expensive than classic in-person therapy, thereby making it more accessible to clients and decreasing the cost (transportation costs, travel time, and missed work). Clients also seem to appreciate the flexibility of teletherapy in terms of where and when they can access treatment	“It is more convenient and it is not like waiting at the office knowing you just have 1 hour to talk.” [59]
	**1.3 Ethical**			
		1.3.1 Privacy	Security. Some clients see teletherapy as more private and secure because they can access it in their homes. This is especially apparent in smaller, tight-knit communities	“Moreover, some service members may be drawn to HBTmental health because of the privacy it offers to those who are concerned about stigma associated with seeking mental health treatment.” [88]
		1.3.2 Safety or risk	Stress. Teletherapy is perceived by some clients as less stressful than in-person therapy. The same clinical skills can be used during remote as in-person delivery for handling heightened emotional responses and symptoms	“Titrating of emotional reactions and patient engagement in traumatic memories, normally including anxiety, increased psychomotor activity, crying, and reexperiencing symptoms, were all handled adequately with the same protocol and clinical skills employed for in-person PE” [58]
	**1.4 Clinical utility**			
		1.4.1 Effectiveness of different types of therapy when delivered via teletherapy	Modalities. Several different evidence-based therapies that have been shown to be effective for teletherapy including prolonged exposure therapy, cognitive processing therapy, and cognitive behavioral therapy	“Use of clinical video teleconferencing services to provide evidence-based treatment to Veterans with posttraumatic stress disorder (PTSD) was found to be as effective as face-to-face treatment provision without negatively impacting therapeutic process measures.” [64]
		1.4.2 Therapy dropout rates in digital health	Dropout. Therapy dropout rates, reasons, and patterns are similar between therapy delivered in-person and remote delivery or therapy dropout rates are dependent more on comorbidities, client life circumstances, and treatment type than on mode of delivery. It is important to note that most of the therapies delivered were based on cognitive behavioral therapy or prolonged exposure therapy	“There were no significant differences in the rates of dropout between the in-person condition and the (home-based) telehealth condition.” [25]

#### Barriers

Multiple barriers were also identified (Table 3), including (1) issues with technology related to connectivity, inconsistent access to a secure, high-quality internet connection, and hardware that disrupts and limits the high quality and secure service delivery [25,52,53,55,64,79,81,85,89], (2) client openness to digital health services [59], (3) challenges to client privacy and comfort, including lack of a quiet, private space, experiences of isolation or disruption in the home environment, and client discomfort with communication over video conferencing [56,59,79,84,85], (4) limits to the therapeutic alliance and therapist comfort with intervention activities that may impact clinical utility and effectiveness [57,58,65,66,79], (5) the ease of abrupt disengagement from treatment and engagement in social avoidant behaviors [55-57,59,79], and (6) safety concerns and risk management [52,59,81].

**Table 3:** Summary of barriers from all studies.

Themes and subthemes		Findings		Illustrative quotes	
**2. Barriers**					
	**2.1 Technological issues are prevalent, causing disruption and limits to high-quality and secure service delivery**				
			Connectivity. Challenges because of problematic client and/or therapist internet connection, video conferencing hardware and software, or problems with server connection commonly present difficulties establishing and maintaining a clear, audible, and uninterrupted video-feed impacts the quality of service delivery and client satisfaction		“The majority of the technical problems that were reported involved lost wireless signals or video or audio quality issues, such as a delay in picture or sound due to poor Internet connection.” [25] “...technical issues with initiating and maintaining a videoconferencing connection were more frequent than expected...” [88]
			Hardware that is compatible for securely connecting with encrypted video conferencing software is not always available for clients. Additionally, as many participants in the studies were provided hardware, more knowledge regarding the protocols and optimal infrastructure for secure delivery of digital health services using personal and/or private computers or video conferencing compatible devices is needed		“An ideal capability would be to use a network infrastructure that meets U.S. Department of Defense network security requirements but that also allows for the use of privately owned end-user equipment (ie, personal computers, Webcams, mobile devices, etc.).” [88]
	**2.2 Perceptions of digital health services may limit client acceptance and openness**				
			Openness to digital health use may depend on previous experiences or recommendations from trusted individuals or sources. Veterans were described as being hesitant to try new technologies because of issues of security or inconsistency with lifestyle (especially in rural populations). As the studies included clients who were seeking services and open to digital health delivery, more knowledge is yet needed of this population’s perceptions and acceptance of digital health services		“Clinical experience, however, suggests that many patients are hesitant to try new technologies.” [55]
	**2.3** **Challenges to client privacy, comfort, and safety exist because of client environment and remote nature of service delivery**				
			Lack of a quiet, private space in which clients can engage in therapy without the fear of being overheard by family members or roommates is common		“... advantages [of digital health] must be balanced by potential shortfalls, such as lack of privacy from family members when televideo sessions are conducted into homes where soundproofing between rooms may not be in place.” [87]
			Session disruptions by doorbells or experiencing an abrupt transition back into everyday life after logging off a session made it difficult to engage from the home environment		“That’s why it was hard to switch from talking all about it and then sort of, the hour’s up and then you’ve got to try and get on with normal life.” [85]
			Some clients indicated discomfort with communication over video despite satisfaction with their therapist. Concerns about managing strong emotions evoked in therapy in an isolated home environment lead clients to prefer in-person treatment. Additionally, clients may be less trusting of the privacy and confidentiality of digital health services		“I do not like not knowing who else is in the room with the therapist.” [55]
			Safety is difficult to manage in a clinically unsupervised environment where a client may be at risk of purposefully terminating a teleconference session while being at risk of suicide. Much of the reviewed literature excluded clients who posed a risk for suicide, and therefore more examples and knowledge on managing risk and responding to a crisis are necessary. Establishing safety protocols involving family members or neighbors and adjusting service delivery schedules to accommodate is a commonly reported measure; feasibility and ethics in doing so must be considered		“Potential drawbacks include... the difficulties of ensuring patient safety in a clinically unsupervised environment.” [55]
	**2.4 Limits to the therapeutic alliance and intervention activities may impact clinical utility and effectiveness**				
			Establishing and building the therapeutic alliance necessary for effective treatment may be challenged because of the impersonal feeling of videoconferencing, which is influenced by an inability to read all the client and therapist nonverbal body cues		“Despite being able to see the therapists face, several participants reported that they felt that doing therapy over Skype felt impersonal because they weren’t in the same room.” [85]
			Therapist comfort with digital health may impact the selection of treatment modalities. Further, some clients may benefit from the in-person presence of a clinician to complete exposure activities as per a treatment protocol. Clients with hypervigilance may be unwilling to close eyes during imaginal exposure as they are not reassured that a therapist can watch out for and respond to threats in their environment. Secure exchange of information online related to intake, assessment, and client homework remains an issue		“Patients who present with more severe symptoms or extreme hypervigilance may be harder to treat via telehealth.” [58]
	**2.5 Ease of disengagement with services and enablement of social avoidant behaviors may be enhanced**				
			Clients can disengage quickly and easily if a session becomes too challenging or uncomfortable. They may engage in distractions during the session, such as watching television or browsing the internet		“if you’re having a bad session, you can just switch him off and walk out the room easily.” [51]
			Enablement of socially avoidant behaviors may occur when delivering mental health service to a client in their home. Care is required to ensure digital health delivery is not discouraging clients from engaging in healthy life events		“...Veterans may require leaving their home and attending face-to-face sessions as part of the therapeutic process.” [59]

#### Recommendations

A number of recommendations were identified to support the use of digital health to deliver evidence-based psychotherapy to military members, veterans, and PSP. Key recommendations included (1) identify and manage technological issues that may impede the use of digital health [25,88], (2) supplement interventions to increase patient comfort [75,87], (3) consider ways to establish and maintain rapport and trust [77,89], (4) be flexible and provide additional support as needed to facilitate progress and commitment to therapy [71,77,85], (5) review previously established standards and practices of delivering certain psychotherapeutic interventions to improve suitability for digital delivery [71,87], (6) address risk and safety issues [88], (7) understand and accommodate demographic factors that can influence the client experience of clients using digital health [62,90], and finally (8) support therapists through training to promote their effective use and uptake of digital health [80,84]. Specific findings related to these key themes are presented in Table 4.

**Table 4:** Recommendations from all studies.

Themes and subthemes			Findings		Illustrative quotes
**3. Recommendations**					
	**3.1 Technological issues that may impede the use of digital health solutions need to be identified and addressed**				
		Backups for information technology disruptions. Service providers need alternatives in place if connectivity issues arise that cannot be resolved through technical assistance from a clinician or technical expert		“If the audio quality remained poor, then the therapist and participant muted their webcams and spoke to each other through the telephone while still using the video feature.” [25]	
		Secure assessments. Secure methods of distributing and collecting assessments and homework assignments need to be considered		“Several modifications were also required for sharing homework and study handouts... such as use of screenshots of homework and handouts and holding handouts up to the camera.” [88]	
	**3.2 Providers can supplement interventions with pretreatment strategies or peer support to increase patients’ comfort with receiving psychotherapy using digital health platforms**				
		Pretreatment strategies can help with preparation for therapy and support participants’ use of digital health		“... the present study incorporated many of the recommendations from Gros and colleagues’ 2013 review, including the preparation session with a walkthrough and testing of the technology, possibly improving the likelihood of acceptance of and satisfaction with telehealth as a result.” [75]	
		Peer assistance can support veterans in becoming more open to digital health and play a role in accomplishing the more difficult aspects of treatment		“... patients who have concerns related to safety or hesitate due to technical concerns may benefit from receiving assistance from a peer before deciding whether or not to try [home telemental health] HTmental health.” [59] “*peer navigators...* may be useful in helping patients to accomplish difficult aspects of treatment, such as in vivo exposure assignments.” [71]	
	**3.3 Providers need to consider ways in which** **rapport and trust can** **be established and maintained between therapists and clients when using digital health**				
		Initial in-person meetings may help to facilitate rapport building for services delivered by digital health		“... simple changes may result in increased adherence to [prolonged exposure therapy] including... meeting the therapist in-person to increase connection and commitment to the treatment provider.” [77]	
		Rapport building. Providers should continue to be mindful about embedding ongoing opportunities within therapy to promote rapport building		“... attention to the development and maintenance of mutually trusting relationships and continued assessment for comfort is recommended.” [89]	
	**3.4 Participants may require additional support and flexibility to support progress and commitment to therapy**				
		Flexible treatment delivery options and additional information before and during therapy, along with practical solutions to support engagement in digital health appointments, can help with progress and commitment to therapy		“... offering a hybrid, in-person + telemedicine option may be useful, and would empower patients to match the modality of treatment delivery to the stage of treatment they are completing.” [71] “... participants reporting that they would have liked to have known more before starting the therapy to better prepare... Many participants said the workbooks and additional information given to them between sessions were beneficial.” [85] “... simple changes may result in increased adherence to [prolonged exposure therapy], including using smart-phone calendar reminders; using personal rather than VA-issued smart-phones...” [77]	
	**3.5 Previously established standards and practices of delivering certain psychotherapeutic interventions need to be analyzed to improve suitability for digital health**				
		Pace of treatment. Due to the independent nature of psychotherapeutic interventions delivered via digital health, there may be a need to alter the pace of treatment		“... increased hyper-vigilance symptoms in telemedicine vs. in-person PTSD treatment groups, may suggest a need for clinical and administrative modifications to the standard exposure therapy protocol when delivered via telemedicine.” [71]	
		Variable intervention delivery. To reduce the likelihood of dropping out of therapy because of temporary symptom exacerbation from exposure exercises, intervention delivery may need to be adjusted (massing sessions at the beginning until benefits are experienced) or adjunct with additional cognitive restructuring exercises or education		“... treatment-interfering cognitions, such as negative treatment expectancies, may need to be addressed with cognitive restructuring in the early stages of treatment.” [57]	
		Addressing avoidance. In patients where digital health may be reinforcing avoidance behaviors, additional education, and discussion to address avoidance behaviors may be warranted. Peer support and encouragement during in vivo exposure exercises may help to reduce the dropout rate		“Veterans engaged in [Clinical Video Technology] CVT-delivered PE or [Cognitive Processing Therapy] may need additional education about the role of avoidance in symptom maintenance and frank discussion about. How the CVT modality may be reinforcing avoidance.” [57]	
	**3.6 Providers need to consider risk and safety of clients because of the remote and independent nature of digital health**				
		Safety planning. Using workable safety standards and planning (including baseline risk assessment, ongoing monitoring of the level of risk, obtaining contact information regarding client's choice of emergency contact before treatment) can facilitate the safe delivery of mental health care to clients in their homes		“All participants completed a release of information form so that a contact person, of their choice, could assist in case of clinical emergency. The requirements and processes for engaging with third parties were disclosed and discussed during the informed consent process.” [88]	
	**3.7 There is a need to recognize and build an understanding of demographic factors (eg, race, gender, and age) that could influence the experience of clients using digital health**				
		Demographic considerations. Recognizing the differences in race and ethnic background for clients should be a priority. Gender, age, and related roles may impact an individual's preference and capacity to receive interventions via digital health; considerations around this should be discussed and reviewed with clients		“Maintaining an understanding of racial obstacles and facilitators in seeking support and continuing follow-up care will be increasingly essential as the military population continues to experience post-traumatic stress related to combat experiences.” [62] “... the effects of age on modality preference among women may reflect barriers to in-office care that uniquely affect the middle-aged and older female populations (eg, responsibility for caring for both older and younger family members, which may make it more difficult to attend office based appointments).” [90] “Younger women may be more likely to have young children in the home, which may require active caregiving during treatment sessions...Therefore, [office-based treatment] may offer a neutral setting where younger women can receive more private care with fewer distractions.” [90]	
	**3.8** **Therapists need to be supported through training to promote effective use and uptake of digital health**				
		Train providers. It is important to support the training of more therapists across a variety of different settings to use digital health to meet the diverse needs of this population		“Given the high amount of turnover and transition among providers within and between deployments, it is imperative that all providers using [clinical videoconferencing] technology be briefed prior to, or at the beginning of, deployment.” [80] “... having multiple providers who can offer [Video To Home] decreases burden of trying to meet diverse needs with only one or two designated providers.” [63]	

## Discussion

### Efficacy of Digital Health Delivery of Trauma Therapies

This review sought to explore, synthesize, and evaluate the available peer-reviewed research regarding needs, gaps, barriers, and facilitators for safe digital health delivery of trauma-focused psychotherapy for military members, veterans, and/or PSP who had a primary diagnosis of PTSD or a stress-related mental health disorder. The review is important and timely given the current COVID-19 pandemic, the concomitant need for physical distancing, and the ongoing need to support those in rural and remote locations, including theaters of operations or war. There is an urgent need to ensure that digital mental health services are accessible, effective, safe, and secure.

Overall, there was encouraging support for the use of digital health to facilitate the treatment of trauma-related mental health problems among military members and veterans. The majority of studies included in this review indicated that digital health was as effective as in-person delivery of psychotherapeutic interventions (CPT, PE, and BA-TE) at clinically and statistically significantly reducing PTSD and depressive symptomatology among military members and veterans with mental health challenges. digital health engagement was not rated by clients as inferior to in-person therapy when it came to building a relationship with their clinician [81]. Interestingly, within the included studies, baseline variables such as age, gender, education level, employment status, and relationship status did not appear to correlate with the effectiveness of the psychotherapeutic intervention or the modality of which it was delivered [63,82,84]. Although results regarding adherence to therapy and dropout varied throughout the studies, the modality of therapy delivery (digital health or in-person) did not appear to be a predictor of adherence or dropout.

Importantly, published work confirms that digital health can improve access to treatment in this population because of a combination of reasons including convenience, cost savings, reduced stigma, and comfort and safety of the home environment. We also note that some studies may have included a selection bias by including participants who were already accepting of the idea of remote delivery of services. In the end, personalization and flexibility of therapy, with attention to multiple overlapping facilitators and barriers, may be required.

### Quality of the Evidence

The quality of the evidence of the studies included in this review was variable and required a review of multiple quantitative, qualitative, and mixed methods methodologies that made data comparisons challenging. The quality of the RCTs included in this study had PEDro scores ranging from *fair* to *excellent*, with the majority of the studies rated as *good* quality. Increased sample sizes within the RCTs would contribute to larger effect sizes and stronger conclusions regarding the efficacy of digital health psychotherapeutic interventions for this population. Although CPT, PE, and BA-TE were rated as level 1a for evidence, there was a multitude of different outcome measures utilized across all studies, which would make it challenging to further complete meta-analyses with conclusive comparisons. The levels of evidence for the utilization of digital health CBT scored *conflicting*, and we noted a variation in results and a lack of consensus. This finding is similar to other published evidence-based literature focusing on generalized trauma-affected populations [91]. The CASP qualitative checklist purposefully does not result in a score for those assessed studies; however, this tool demonstrated that the qualitative studies were of sound methodology and added a valuable contribution to the literature. Finally, the literature base is relatively new, and as such, it is still fundamentally limited with studies predominantly focusing on 3 therapeutic modalities: PE, CPT, and CBT interventions. Other efficacious psychotherapeutic interventions would benefit from studies with military members, veterans, and/or PSP.

### Facilitators, Barriers, and Recommendations

Facilitators, barriers, and recommendations reported in the literature (Tables 2, 3 and 4, respectively) offer clinical and research community insights into ways to respond to the urgent need to research and remotely deliver mental health services. In particular, identified facilitators need to be maximized, barriers overcome, and recommendations implemented for digital health to be best used in practice; doing so will require policy, practice, and system change. As digital health delivery can result in more timely help-seeking behaviors by military members, veterans, and/or PSP, it would be beneficial for more mental health clinicians and mental health clinics to incorporate digital health delivery options as one of several modes of service delivery routinely available to clients [29,31]. This may involve complementing in-person with digital service delivery and varying hours of access to services beyond traditional clinical hours of operation. Doing so would require mental health clinicians and clients to have appropriate technologies, have access to systems and supports, and have strategies and policies in place to ensure connectivity, privacy, security, and confidentiality [84].

Providing immediate support to military members, veterans, and PSP through digital means, particularly when they are contending with operational stresses and trauma, may reduce the acute and long-term impacts on military members, veterans, and PSP themselves and on their teams, organizations, and families [81,89]. Digitally connecting mental health clinicians and remotely or rurally located clients can result in military members, veterans, and PSP receiving support and services that they might not receive otherwise [29,31,55].

The benefits of timely and responsive interventions can reach beyond the individual and into family, organization, and community life [89]. This can positively impact the well-being, operational readiness, and pandemic response. It can also potentially better serve those who might not otherwise seek treatment such as disadvantaged or minority groups, or those who have experienced sexual trauma or operational stress injuries, for example [55,57,59,62,81].

Although the literature regarding the use of digital health to deliver trauma therapies is encouraging, some specific concerns regarding remote delivery warrant consideration. Receiving therapy in one’s home, for example, may increase the likelihood of military members, veterans, and PSP avoiding traumatic cues and/or engaging in avoidance behaviors, although this may be more of a dialectic than an actual impediment. That being said, starting with digital health may enable those who would otherwise find it difficult to engage and face their anxieties to actually do so. This may be analogous to the difference between flooding and gradual, systematic desensitization, where digital health may represent the first step for some clients toward challenging, more difficult avoidance behaviors outside the home environment. Similar to the risk management strategies required for in-person service delivery with military members, veterans, and PSP who are at increased risk of self-harm, violence, and suicidality, safeguards need to be implemented when using digital health–delivered psychotherapeutic interventions. It is important to note that the reviewed literature does not indicate a heightened risk of suicide per se associated with digital health interventions. Consequently, while safety planning is as important for digital health as it is for in-person therapy, suicide risk should not automatically preclude the digital delivery of mental health interventions [76]. Finally, not all patients benefit equally from the digital health interventions. Whereas engaging in therapy at home can be a facilitator for certain clients, some may experience traumatic cues, interruptions, be concerned about privacy, or have discomfort in revealing their home environment to the therapist [77]. This speaks to the importance of determining the right therapy and mode of delivery for the right person at the right time.

Although this scoping review was specific to digital health interventions specific to military member, veterans, and PSP, it may also assist clinicians and researchers in the use of digital health in the general civilian population. It will also add to the growing body of literature exploring digital health utilization for other conditions (ie, substance use, anxiety, depression, dual-diagnosis, schizophrenia, cancer, chronic pain).

### Knowledge Gaps and Future Research

With digital health interventions being a relatively recent necessity as a result of the COVID-19 pandemic, this review highlights key gaps in the peer-reviewed literature and areas requiring future research. With regard to user factors, there is a lack of research (ie, only 3 identified studies) related specifically to PSP, emphasizing the need for further study of digital health use in this population. In addition, the majority of studies were conducted in the United States, pointing to the need for future studies to be conducted with military members, veterans, and/or PSP from other international jurisdictions. Further work is also needed not only regarding the impact of demographic and contextual factors but also on the client’s stage of treatment, level of functioning, illness severity, attachment style, and comorbidities. How common comorbidities such as depression, personality disorders, or dissociation impact engagement or outcomes in digital health is also yet unknown. For example, some patients require more coregulation in session with the therapist, and there is little information about how digital health delivery might impact this process.

Factors specific to mental health clinicians also warrant further study. This includes consideration of their attitudes, technology acceptance, and usability of digital health. Moreover, little is known about the clinicians’ concerns about the impact of digital health on safety, risk management, and control of the therapeutic situation. It is currently unclear whether a clinician’s comfort and belief in the efficacy of digital health interventions has a similar influence on therapeutic outcome. It is also important to understand the training and support needs of clinicians to adapt previous in-person interventions to digital health delivery formats. There are also practical aspects to consider regarding the delivery of therapy via digital health. For example, it is currently not well known how the prolonged use of videoconferencing technologies affects a clinician’s level of fatigue, attention, and level of engagement throughout a session.

Therapeutic factors associated with the use of digital health also warrant further investigation. This includes factors associated with establishing and maintaining the therapeutic relationship between the clinician and client to those around safety and containment. Similarly, types of treatments that lend themselves better to digital delivery need to be determined and studied. Some therapeutic modalities, for example, are more dependent on in-person therapeutic relational factors (eg, the ability to read body language), whereas others involve experiential activities that may prove difficult to duplicate in a remote delivery context, particularly in the absence of secure platforms to facilitate homework and other barriers. CBT and digital health delivery for military members, veterans, and PSP also warrant additional high-quality research to address the current conflict in the literature. Furthermore, the use of digital health for the delivery of group-based interventions also warrants further study, specifically as it relates to group dynamics, privacy, and stigma, and particularly given the benefits and potential cost-efficiency of group interventions. Future research into individualization of digital health therapy, with attention to multiple facilitators and barriers, is required. Finally, at the macrolevel, further research is needed to determine how to best address systemic issues such as health system policies and medicolegal issues that may present as barriers to digital health for health care organizations, administrators, managers, mental health clinicians, and, therefore, the clients they serve.

### Strengths and Limitations

Significant efforts were made to ensure the rigor and quality of the review. Notably, it was performed following a priori and detailed procedures with increased attention to ensure quality control and reduce bias [41,42]. Furthermore, the search strategy was extensive and included 5 databases. Inclusion and exclusion criteria were determined before study onset and adhered to throughout the study. Appropriate calibration and pilot testing, use of at least two independent reviewers for all stages of the process, additional verification of extracted data, and group discussion of conflicts improved the quality of the review.

The authors also acknowledge several limitations of this review. First, the review specifically selected articles focusing on military members, veterans, and/or PSP who had a primary diagnosis of PTSD or a stress-related mental health disorder. Thus, there may be additional informative literature that focuses on other populations. Second, with an imposed date limit, it is possible that quality studies published before 2010 may have been missed; however, with the rapid speed of advancement in digital health, technology, access to technology, and its quality, it is unlikely that research on technology from more than a decade ago before the widespread use of smartphones holds exceptional relevance to this review. Finally, there are limits to aggregate data.

### Conclusions

In light of COVID-19, research regarding the remote delivery of trauma therapies for military members, veterans, and PSP is no longer simply novel, but a real-world necessity. In addition, during times of physical distancing, digital health is a mode of delivering psychotherapeutic interventions for those who are in remote locations, including rural areas or during tactical and operational deployment. Despite some promising evidence in currently published literature, health care organizations and mental health clinicians should continue to proceed cautiously with remote delivery of psychotherapeutic trauma therapies as much research is still needed to address *the digital divide* among trauma-affected military members, veterans, and/or PSP. This systematic scoping review included 38 studies researching factors related to the use of digital health to deliver psychotherapeutic interventions for military members, veterans, and/or PSP affected by trauma. Evidence for the effectiveness of digital delivery of PE, CPT, and BA-TE on military members, veterans, and PSP was rated level 1a, whereas evidence for CBT was conflicting. The narrative synthesis suggests that digital health delivery of these therapies can be as effective as in-person delivery; for some, it may increase access, reduce stigma, and facilitate engagement with mental health clinicians. Issues of risk, safety, privacy and security, digital health modality, clinician factors, and barriers to digital health need to be researched further as does the potential for additional trauma therapies, including ART, ADT, and EMDR, to be suitable for digital delivery. Further elucidation is also needed for gender, racial, and cultural factors that may create differences in client outcomes, preferences, and needs. Professional organizations are likely needed to invest in training for clinicians, formulate guidelines for digital health delivery of trauma therapies, formulate ethical and medicolegal guidance, and assist in adapting to the digital health environment.

## Appendix

Multimedia Appendix 1 Detailed search strategy.

Multimedia Appendix 2 Detailed descriptive analysis of studies included in the review.

Multimedia Appendix 3 Descriptive analysis and outcomes of all studies included in the scoping review.

## Abbreviations

ADT: adaptive disclosure therapy
ART: accelerated resolution therapy
BA: behavioral activation
BA-TE: behavioral activation and therapeutic exposure
CASP: Critical Skills Appraisal Program
CBT: cognitive behavioral therapy
CPT: cognitive processing therapy
EMDR: eye movement desensitization reprocessing
PE: prolonged exposure therapy
PEDro: Physiotherapy Evidence Database
PRISMA: Preferred Reporting Items for Systematic Reviews and Meta-Analyses
PSP: public safety personnel
PTSD: posttraumatic stress disorder
PTSI: posttraumatic stress injury
RCT: randomized controlled trial
