# Supplementary material for: Virtual Trauma-Focused Therapy for Military Members, Veterans, and Public Safety Personnel With Posttraumatic Stress Injury: Systematic Scoping Review
Source: JMIR Mhealth Uhealth. 2020 Sep 21;8(9):e22079. doi: 10.2196/22079 (PMC7536597; doi:10.2196/22079)
Supplement: Multimedia Appendix 1 [file mhealth_v8i9e22079_app1.docx]

## Appendix A

### Detailed Search Strategy

#### Ovid MEDLINE(R) ALL 1946 to May 28, 2020

Results: 173

Date searched: May 29, 2020

1. stress disorders, traumatic/ or Stress Disorders, Traumatic, Acute/ or combat disorders/ or psychological trauma/ or stress disorders, post-traumatic/

2. (PTSD or PTSI or PTSS or OSI or ((posttraumatic or post traumatic or combat or war or trauma* or operational) adj1 (growth or stress* or neurosis or neuroses or nightmare*)) or ((traumatic or acute) adj (stress disorder* or stress symptom*)) or shell shock* or shellshock* or (vicarious* adj2 trauma*)).mp.

3. (suicid* or self-harm* or psychological* trauma* or emotional* trauma* or Mental Health Injur* or Psychological* Injur* or Mental Health Condition* or Mental Health Challeng* or mental* ill* or (mental adj2 disorder*)).mp.

4. mental disorders/ or exp substance-related disorders/ or "trauma and stressor related disorders"/

5. Behavior, Addictive/

6. (((Substance or cocaine or opioid* or performance enhanc* or cannabis or marijuana or alcohol) adj3 (abuse or "use" or misuse)) or addiction*).mp.

7. (((moral* or ethical) adj4 (injur* or distress*)) or "sanctuary trauma" or complex grief or complicated bereavement or complicated grief).mp.

8. ((organizational or institutional) adj4 (betrayal or trauma*)).mp.

9. or/1-8

10. Military Personnel/

11. Military Medicine/

12. Rescue Work/

13. Emergency Medical Dispatch/

14. exp Emergency Responders/

15. (combat fatigue or combat disorder* or Military or paramilitary or armed-force* or veteran* or armed-service* or servicewomen or servicemen or air-personnel or defense-force* or defence-force* or service-personnel or army or navy or air-force or marine* or sailor* or soldier* or infantryman or Civil-defense or Troops or ranger* or "medic" or coast guard or submariner* or active duty or enlisted personnel or reserve personnel or police* or RCMP or officer* or firefighter* or firem* or EMT or EMTs or EMS or (Emergency Medical adj2 (personnel or technician*)) or paramedic* or (public safety adj3 (professional* or official* or personnel*)) or "first responder*" or "law enforcement" or "search and rescue" or "medic" or medics or ambulance or ((corrections or correctional) adj (worker* or staff or personnel or official*)) or security guard* or security personnel or sheriff* or (border adj3 (agent* or personnel or security)) or emergency manager* or coms or comms or communication-official* or (("911" or emergency) adj3 (dispatcher or personnel)) or intel or (intelligence adj3 (personnel or agent*))).mp.

16. or/10-15

17. telemedicine/ or remote consultation/ or telerehabilitation/

18. Videoconferencing/

19. (Telepsychiatr* or teletherap* or telerehab* or tele-rehab* or telehealth or telemedicine or teleconsult* or mobile-health* or mhealth or m-health or distance health* or electronic-health* or ehealth or e-health or virtual health or Remote delivery or electronic-delivery or digital health or video conferenc* or videoconferenc* or teleconferenc* or tele-conferen* or video-to-home or video-visit* or video-technology or skype or google meet or zoom).mp.

20. ((Online or virtual or remote* or video or digital or telephone or phone or tele) adj4 (Psychotherap* or psychiatr* or therapy or treatment* or counseling or intervention* or program* or diagnos* or assessment or evaluation or follow-up or care or health or consult* or communicat* or medicine)).mp.

21. ((remote or online or virtual* or electronic* or digital* or distan*) adj2 deliver*).mp.

22. ((remote or online or virtual* or electronic* or digital* or distan* or video or tele or phone or telephone) adj10 (motivational interviewing or cbt or behavio* therap* or Cognitive Processing Therapy or "Eye movement desensitization and reprocessing" or EMDR or CPT or exposure therap* or prolonged exposure or accelerated resolution therap* or adaptive disclosure therap* or ADP or (trauma-informed adj3 (care or approach* or therap* or treatment* or intervention*)))).mp.

23. or/17-22

24. (motivational interviewing or cbt or behavio* therap* or Cognitive Processing Therapy or "Eye movement desensitization and reprocessing" or EMDR or CPT or exposure therap* or prolonged exposure or accelerated resolution therap* or adaptive disclosure therap* or ADP or (trauma-informed adj3 (care or approach* or therap* or treatment* or intervention*))).mp.

25. 9 and 16 and 23 and 24

26. limit 25 to yr="2010 -Current"

27. limit 26 to english language

28. limit 27 to (comment or editorial or historical article or interview or lecture or news or newspaper article or observational study, veterinary or personal narrative)

29. 27 not 28

#### Embase 1974 to 2020 May 28

Results:185

Date searched: May 29, 2020

1. posttraumatic stress disorder/ or acute stress disorder/

2. (PTSD or PTSI or PTSS or OSI or ((posttraumatic or post traumatic or combat or war or trauma* or operational) adj1 (growth or stress* or neurosis or neuroses or nightmare*)) or ((traumatic or acute) adj (stress disorder* or stress symptom*)) or shell shock* or shellshock* or (vicarious* adj2 trauma*)).mp.

3. (suicid* or self-harm* or Psychological* Trauma* or emotional* trauma* or Mental Health Injur* or Psychological* Injur* or Mental Health Condition* or Mental Health Challeng* or mental* ill* or (mental adj2 disorder*)).mp.

4. mental disease/ or addiction/ or complicated grief/ or exp psychotrauma/

5. exp behavioral addiction/ or exp drug dependence/

6. (((Substance or cocaine or opioid* or performance enhanc* or cannabis or marijuana or alcohol) adj3 (abuse or "use" or misuse)) or addiction*).mp.

7. substance abuse/

8. (((moral* or ethical) adj4 (injur* or distress*)) or "sanctuary trauma" or complex grief or complicated bereavement or complicated grief).mp.

9. ((organizational or institutional) adj4 (betrayal or trauma*)).mp.

10. or/1-9

11. army/

12. military medicine/

13. military personnel/

14. rescue personnel/

15. fire fighter/ or exp police/ or veteran/

16. (combat fatigue or combat disorder* or Military or paramilitary or armed-force* or veteran* or armed-service* or servicewomen or servicemen or air-personnel or defense-force* or defence-force* or service-personnel or army or navy or air-force or marine* or sailor* or soldier* or infantryman or Civil-defense or Troops or ranger* or "medic" or coast guard or submariner* or active duty or enlisted personnel or reserve personnel or police* or RCMP or officer* or firefighter* or firem* or EMT or EMTs or EMS or (Emergency Medical adj2 (personnel or technician*)) or paramedic* or (public safety adj3 (professional* or official* or personnel*)) or "first responder*" or "law enforcement" or "search and rescue" or "medic" or medics or ambulance or ((corrections or correctional) adj (worker* or staff or personnel or official*)) or security guard* or security personnel or sheriff* or (border adj3 (agent* or personnel or security)) or emergency manager* or coms or comms or communication-official* or (("911" or emergency) adj3 (dispatcher or personnel)) or intel or (intelligence adj3 (personnel or agent*))).mp.

17. or/11-16

18. teleconsultation/

19. telehealth/ or telenursing/ or telemedicine/ or teleconsultation/ or telediagnosis/ or telemonitoring/ or telepsychiatry/ or telerehabilitation/ or teletherapy/

20. videoconferencing/

21. (Telepsychiatr* or teletherap* or telerehab* or tele-rehab* or telehealth or telemedicine or teleconsult* or mobile-health* or mhealth or m-health or distance health* or electronic-health* or ehealth or e-health or virtual health or Remote delivery or electronic-delivery or digital health or video conferenc* or videoconferenc* or teleconferenc* or tele-conferen* or video-to-home or video-visit* or video-technology or zoom or google meet or skype).mp.

22. ((Online or virtual or remote* or video or digital or telephone or phone or tele) adj4 (Psychotherap* or psychiatr* or therapy or treatment* or counseling or intervention* or program* or diagnos* or assessment or evaluation or follow-up or care or health or consult* or communicat* or medicine)).mp.

23. ((remote or online or virtual* or electronic* or digital* or distan*) adj2 deliver*).mp.

24. ((remote or online or virtual* or electronic* or digital* or distan* or video or tele or phone or telephone) adj10 (motivational interviewing or cbt or behavio* therap* or Cognitive Processing Therapy or "Eye movement desensitization and reprocessing" or EMDR or CPT or exposure therap* or prolonged exposure or accelerated resolution therap* or adaptive disclosure therap* or ADP or (trauma-informed adj3 (care or approach* or therap* or treatment* or intervention*)))).mp.

25. or/18-24

26. (motivational interviewing or cbt or behavio* therap* or Cognitive Processing Therapy or "Eye movement desensitization and reprocessing" or EMDR or CPT or exposure therap* or prolonged exposure or accelerated resolution therap* or adaptive disclosure therap* or ADP or (trauma-informed adj3 (care or approach* or therap* or treatment* or intervention*))).mp.

27. 10 and 17 and 25 and 26

28. limit 27 to yr="2010 -Current"

29. limit 28 to conference abstracts

30. 28 not 29

31. limit 30 to (conference abstract or conference paper or "conference review" or editorial)

32. 30 not 31

#### APA PsycInfo 1806 to May Week 4 2020

Results: 179

Date searched: May 29, 2020

1. "stress and trauma related disorders"/ or exp posttraumatic stress disorder/ or post-traumatic stress/ or acute stress disorders/

2. emotional trauma/

3. "substance related and addictive disorders"/ or exp nonsubstance related addictions/ or exp "substance use disorder"/ or addiction treatment/

4. chronic mental illness/

5. mental disorders/

6. (PTSD or PTSI or PTSS or OSI or ((posttraumatic or post traumatic or combat or war or trauma* or operational) adj1 (growth or stress* or neurosis or neuroses or nightmare*)) or ((traumatic or acute) adj (stress disorder* or stress symptom*)) or shell shock* or shellshock* or (vicarious* adj2 trauma*)).mp.

7. (suicid* or self-harm* or Psychological* Trauma* or emotional* trauma* or Mental Health Injur* or Psychological* Injur* or Mental Health Condition* or Mental Health Challeng* or mental* ill* or (mental adj2 disorder*)).mp.

8. (((Substance or cocaine or opioid* or performance enhanc* or cannabis or marijuana or alcohol) adj3 (abuse or "use" or misuse)) or addiction*).mp.

9. (((moral* or ethical) adj4 (injur* or distress*)) or "sanctuary trauma" or complex grief or complicated bereavement or complicated grief).mp.

10. ((organizational or institutional) adj4 (betrayal or trauma*)).mp.

11. or/1-10

12. exp military personnel/ or combat experience/ or military psychiatry/

13. exp emergency personnel/

14. (combat fatigue or combat disorder* or Military or paramilitary or armed-force* or veteran* or armed-service* or servicewomen or servicemen or air-personnel or defense-force* or defence-force* or service-personnel or army or navy or air-force or marine* or sailor* or soldier* or infantryman or Civil-defense or Troops or ranger* or "medic" or coast guard or submariner* or active duty or enlisted personnel or reserve personnel or police* or RCMP or officer* or firefighter* or firem* or EMT or EMTs or EMS or (Emergency Medical adj2 (personnel or technician*)) or paramedic* or (public safety adj3 (professional* or official* or personnel*)) or "first responder*" or "law enforcement" or "search and rescue" or "medic" or medics or ambulance or ((corrections or correctional) adj (worker* or staff or personnel or official*)) or security guard* or security personnel or sheriff* or (border adj3 (agent* or personnel or security)) or emergency manager* or coms or comms or communication-official* or (("911" or emergency) adj3 (dispatcher or personnel)) or intel or (intelligence adj3 (personnel or agent*))).mp.

15. or/12-14

16. exp telemedicine/

17. digital interventions/

18. videoconferencing/

19. video-based interventions/

20. (Telepsychiatr* or teletherap* or telerehab* or tele-rehab* or telehealth or telemedicine or teleconsult* or mobile-health* or mhealth or m-health or distance health* or electronic-health* or ehealth or e-health or virtual health or Remote delivery or electronic-delivery or digital health or video conferenc* or videoconferenc* or teleconferenc* or tele-conferen* or video-to-home or video-visit* or video-technology or zoom or google meet* or skype).mp.

21. ((Online or virtual or remote* or video or digital or telephone or phone or tele) adj4 (Psychotherap* or psychiatr* or therapy or treatment* or counseling or intervention* or program* or diagnos* or assessment or evaluation or follow-up or care or health or consult* or communicat* or medicine)).mp.

22. ((remote or online or virtual* or electronic* or digital* or distan*) adj2 deliver*).mp.

23. ((remote or online or virtual* or electronic* or digital* or distan* or video or tele or phone or telephone) adj10 (motivational interviewing or cbt or behavio* therap* or Cognitive Processing Therapy or "Eye movement desensitization and reprocessing" or EMDR or CPT or exposure therap* or prolonged exposure or accelerated resolution therap* or adaptive disclosure therap* or ADP or (trauma-informed adj3 (care or approach* or therap* or treatment* or intervention*)))).mp.

24. or/16-23

25. (motivational interviewing or cbt or behavio* therap* or Cognitive Processing Therapy or "Eye movement desensitization and reprocessing" or EMDR or CPT or exposure therap* or prolonged exposure or accelerated resolution therap* or adaptive disclosure therap* or ADP or (trauma-informed adj3 (care or approach* or therap* or treatment* or intervention*))).mp.

26. 11 and 15 and 24 and 25

27. limit 26 to yr="2010 -Current"

28. limit 27 to english language

29. limit 28 to ("column/opinion" or dissertation or editorial or encyclopedia entry or interview or obituary or poetry or review-book or review-media or review-software & other)

30. 28 not 29

#### CINAHL PLUS with Full Text (EBSCOhost interface)

Results: 72

Date searched: May 29, 2020

Deselect: Apply Equivalent Subjects (for ALL lines)

S1. (MH "Stress Disorders, Post-Traumatic") OR (MH "Psychological Trauma") OR (MH "Behavior, Addictive+") OR (MH "Substance Dependence+") OR (MH "Substance Abuse") OR (MH "Alcohol-Related Disorders+") OR (MH "Inhalant Abuse") OR (MH "Substance Abuse, Intravenous") OR (MH "Mental Disorders, Chronic") OR (MH "Mental Disorders")

S2. PTSD or PTSI or PTSS or OSI or ((posttraumatic or post-traumatic or combat or war or trauma* or operational) N1 (growth or stress* or neurosis or neuroses or nightmare*)) or ((traumatic or acute) N1 (stress disorder* or stress symptom*)) or shell-shock* or shellshock* or (vicarious* N2 trauma*) OR psychological*-trauma* or Mental-Health-Injur* or suicid* or self-harm* or Psychological*-Injur* or Mental-Health-Condition* or Mental-Health-Challeng* or mental*-ill* or mental-disorder* OR mental-health-disorder* or addiction* or ((Substance or cocaine or opioid* or performance enhanc* or cannabis or marijuana or alcohol) N3 (abuse or "use" or misuse)) OR ((moral* or ethical) N4 (injur* or distress*)) or "sanctuary trauma" or complex-grief or complicated-bereavement or complicated-grief OR ((organizational or institutional) N4 (betrayal or trauma*))

S3. S1 OR S2

S4. ( (MH "Military Services+") OR (MH "Military Medicine") OR (MH "Military Personnel+") OR (MH "Military Nursing") OR (MH "Military Training") OR (MH "Military Recruits") OR (MH "Military Deployment") OR (MH "Firefighters") OR (MH "Police") OR (MH "Emergency Medical Technicians") ) OR ( combat-fatigue or combat-disorder* or Military or paramilitary or armed-force* or veteran* or armed-service* or servicewomen or servicemen or air-personnel or defense-force* or defence-force* or service-personnel or army or navy or air-force or marine* or sailor* or soldier* or infantryman or Civil-defense or Troops or ranger* or "medic" or coast guard or submariner* or active duty or enlisted personnel or reserve personnel or police* or RCMP or officer* or firefighter* or firem* or EMT or EMTs or EMS or (Emergency Medical N2 (personnel or technician*)) or paramedic* or (public safety N3 (professional* or official* or personnel*)) or "first responder*" or "law enforcement" or "search and rescue" or "medic" or medics or ambulance or ((corrections or correctional) N1 (worker* or staff or personnel or official*)) or security-guard* or security-personnel or sheriff* or (border N3 (agent* or personnel or security)) or emergency manager* or coms or comms or communication-official* or (("911" or emergency) N3 (dispatcher or personnel)) or intel or (intelligence N3 (personnel or agent*)) )

S5. ( (MH "Telehealth") OR (MH "Telemedicine") OR (MH "Remote Consultation") OR (MH "Telerehabilitation") OR (MH "Telenursing") OR (MH "Telepsychiatry") OR (MH "Videoconferencing") ) OR ( Telepsychiatr* or teletherap* or telerehab* or tele-rehab* or telehealth or telemedicine or teleconsult* or mobile-health* or mhealth or m-health or distance health* or electronic-health* or ehealth or e-health or virtual-health or Remote-delivery or electronic-delivery or digital-health or video-conferenc* or videoconferenc* or teleconferenc* or tele-conferen* or video-to-home or video-visit* or video-technology OR skype or google-meet or zoom or ((Online or virtual or remote* or video or digital or telephone or phone or tele) N4 (Psychotherap* or psychiatr* or therapy or treatment* or counseling or intervention* or program* or diagnos* or assessment or evaluation or follow-up or care or health or consult* or communicat* or medicine)) OR ((remote or online or virtual* or electronic* or digital* or distan*) N2 deliver*) ) OR ((remote or online or virtual* or electronic* or digital* or distan* or video or tele or phone or telephone) N10 (motivational interviewing or cbt or behavio* therap* or Cognitive Processing Therapy or "Eye movement desensitization and reprocessing" or EMDR or CPT or exposure therap* or prolonged exposure or accelerated resolution therap* or adaptive disclosure therap* or ADP or (trauma-informed N3 (care or approach* or therap* or treatment* or intervention*))))

S6. (motivational interviewing or cbt or behavio* therap* or Cognitive Processing Therapy or "Eye movement desensitization and reprocessing" or EMDR or CPT or exposure therap* or prolonged exposure or accelerated resolution therap* or adaptive disclosure therap* or ADP or (trauma-informed N3 (care or approach* or therap* or treatment* or intervention*)))

S7. S3 AND S4 AND S5 Limiters - Scholarly (Peer Reviewed) Journals; English Language; Published Date: 20100101-20201231

#### Military and Government Collection (EBSCOhost interface)

Results: 20

Date searched: May 29, 2020

Deselect: Apply Equivalent Subjects (for ALL lines)

S1 PTSD or PTSI or PTSS or OSI or ((posttraumatic or post-traumatic or combat or war or trauma* or operational) N1 (growth or stress* or neurosis or neuroses or nightmare*)) or ((traumatic or acute) N1 (stress disorder* or stress symptom*)) or shell-shock* or shellshock* or (vicarious* N2 trauma*) OR suicid* or self-harm* or psychological*-trauma* or emotional*-trauma* or Mental-Health-Injur* or Psychological*-Injur* or Mental-Health-Condition* or Mental-Health-Challeng* or mental*-ill* or mental-disorder* OR mental-health-disorder* or addiction* or ((Substance or cocaine or opioid* or performance enhanc* or cannabis or marijuana or alcohol) N3 (abuse or "use" or misuse)) OR ((moral* or ethical) N4 (injur* or distress*)) or "sanctuary trauma" or complex-grief or complicated-bereavement or complicated-grief OR ((organizational or institutional) N4 (betrayal or trauma*))

S2. ( Telepsychiatr* or teletherap* or telerehab* or tele-rehab* or telehealth or telemedicine or teleconsult* or mobile-health* or mhealth or m-health or distance health* or electronic-health* or ehealth or e-health or virtual-health or Remote-delivery or electronic-delivery or digital-health or video-conferenc* or videoconferenc* or teleconferenc* or tele-conferen* or video-to-home or video-visit* or video-technology OR ((Online or virtual or remote* or video or digital or telephone or phone or tele) N4 (Psychotherap* or psychiatr* or therapy or treatment* or counseling or intervention* or program* or diagnos* or assessment or evaluation or follow-up or care or health or consult* or communicat* or medicine)) OR ((remote or online or virtual* or electronic* or digital* or distan*) N2 deliver*) ) OR ((remote or online or virtual* or electronic* or digital* or distan* or video or tele or phone or telephone) N10 (motivational interviewing or cbt or behavio* therap* or Cognitive Processing Therapy or "Eye movement desensitization and reprocessing" or EMDR or CPT or exposure therap* or prolonged exposure or accelerated resolution therap* or adaptive disclosure therap* or ADP or (trauma-informed N3 (care or approach* or therap* or treatment* or intervention*))))

S3. combat-fatigue or combat-disorder* or Military or paramilitary or armed-force* or veteran* or armed-service* or servicewomen or servicemen or air-personnel or defense-force* or defence-force* or service-personnel or army or navy or air-force or marine* or sailor* or soldier* or infantryman or Civil-defense or Troops or ranger* or "medic" or coast guard or submariner* or active duty or enlisted personnel or reserve personnel or police* or RCMP or officer* or firefighter* or firem* or EMT or EMTs or EMS or (Emergency Medical N2 (personnel or technician*)) or paramedic* or (public safety N3 (professional* or official* or personnel*)) or "first responder*" or "law enforcement" or "search and rescue" or "medic" or medics or ambulance or ((corrections or correctional) N1 (worker* or staff or personnel or official*)) or security-guard* or security-personnel or sheriff* or (border N3 (agent* or personnel or security)) or emergency manager* or coms or comms or communication-official* or (("911" or emergency) N3 (dispatcher or personnel)) or intel or (intelligence N3 (personnel or agent*))

S4: (motivational interviewing or cbt or behavio* therap* or Cognitive Processing Therapy or "Eye movement desensitization and reprocessing" or EMDR or CPT or exposure therap* or prolonged exposure or accelerated resolution therap* or adaptive disclosure therap* or ADP or (trauma-informed N3 (care or approach* or therap* or treatment* or intervention*)))

S5. S1 AND S2 AND S3 AND S4

Limiters - Scholarly (Peer Reviewed) Journals; Published Date: 20100101-20201231
