# Supplementary material for: Virtual Trauma-Focused Therapy for Military Members, Veterans, and Public Safety Personnel With Posttraumatic Stress Injury: Systematic Scoping Review
Source: JMIR Mhealth Uhealth. 2020 Sep 21;8(9):e22079. doi: 10.2196/22079 (PMC7536597; doi:10.2196/22079)

## Appendix B: Detailed Descriptive Analysis of Studies Included in Review

### Figure 1: Year of Article Publication (n= 38)


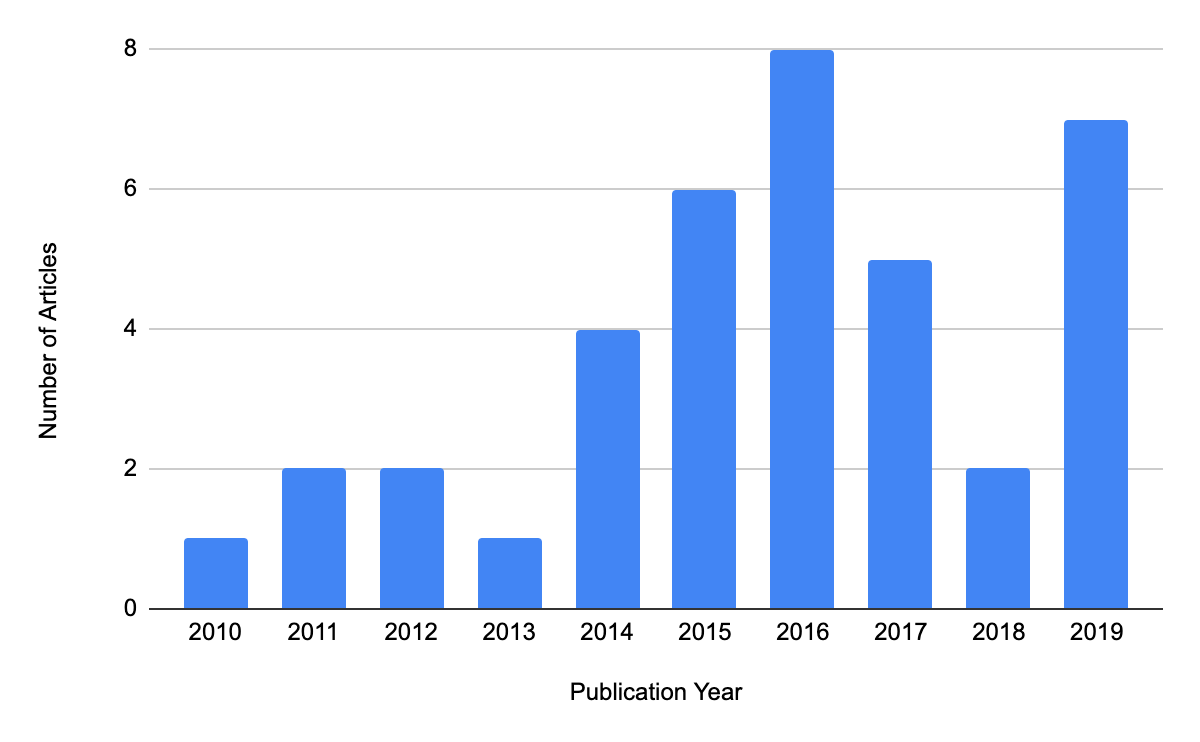


Figure 2: Country of Authors (n= 38)


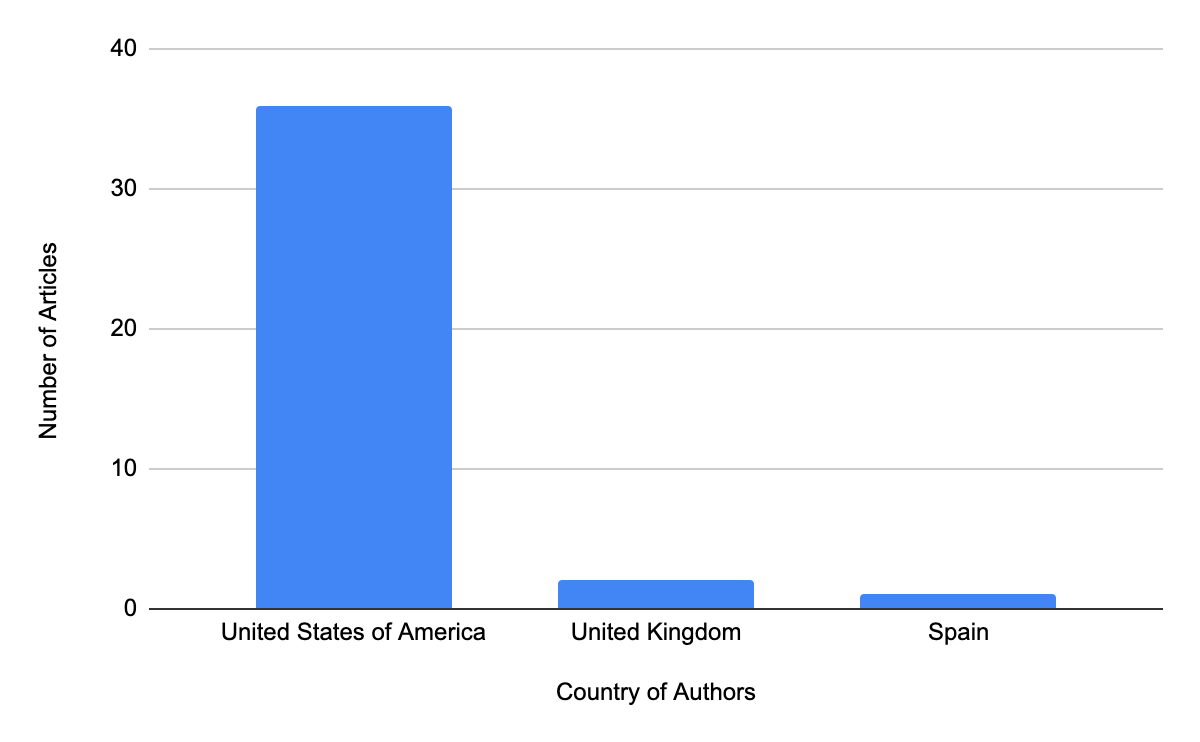


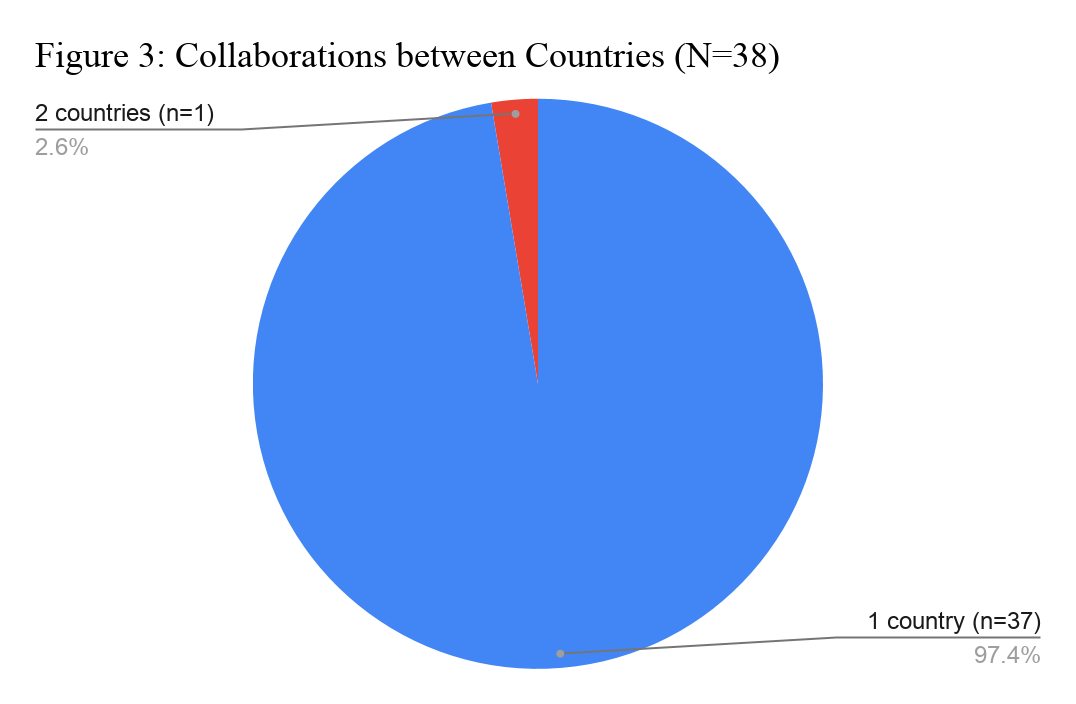

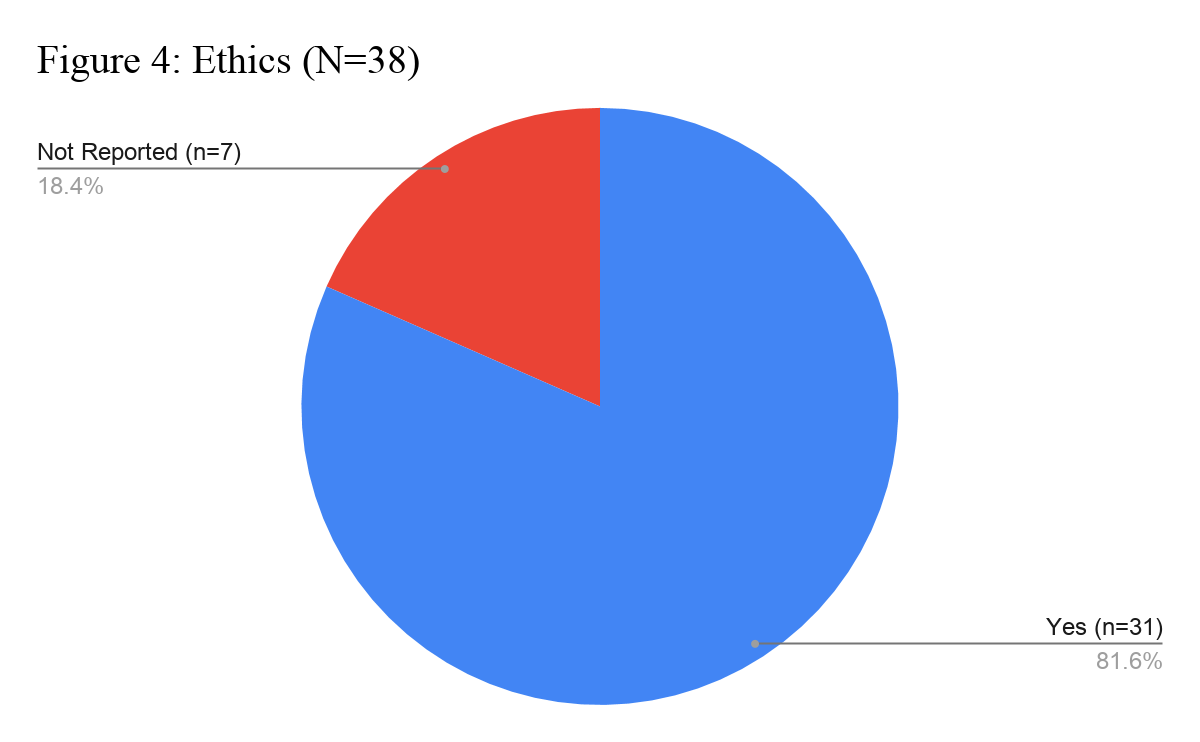

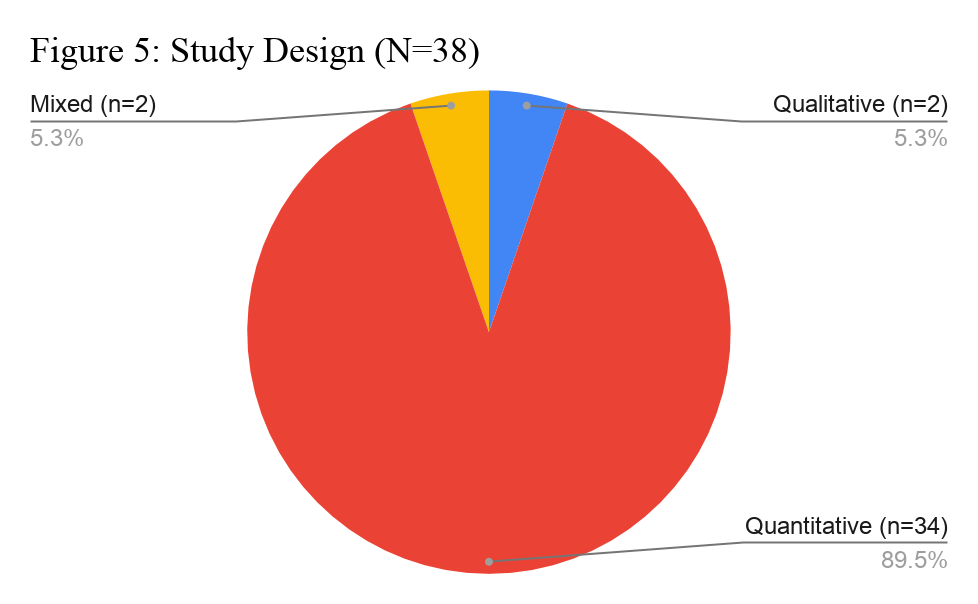


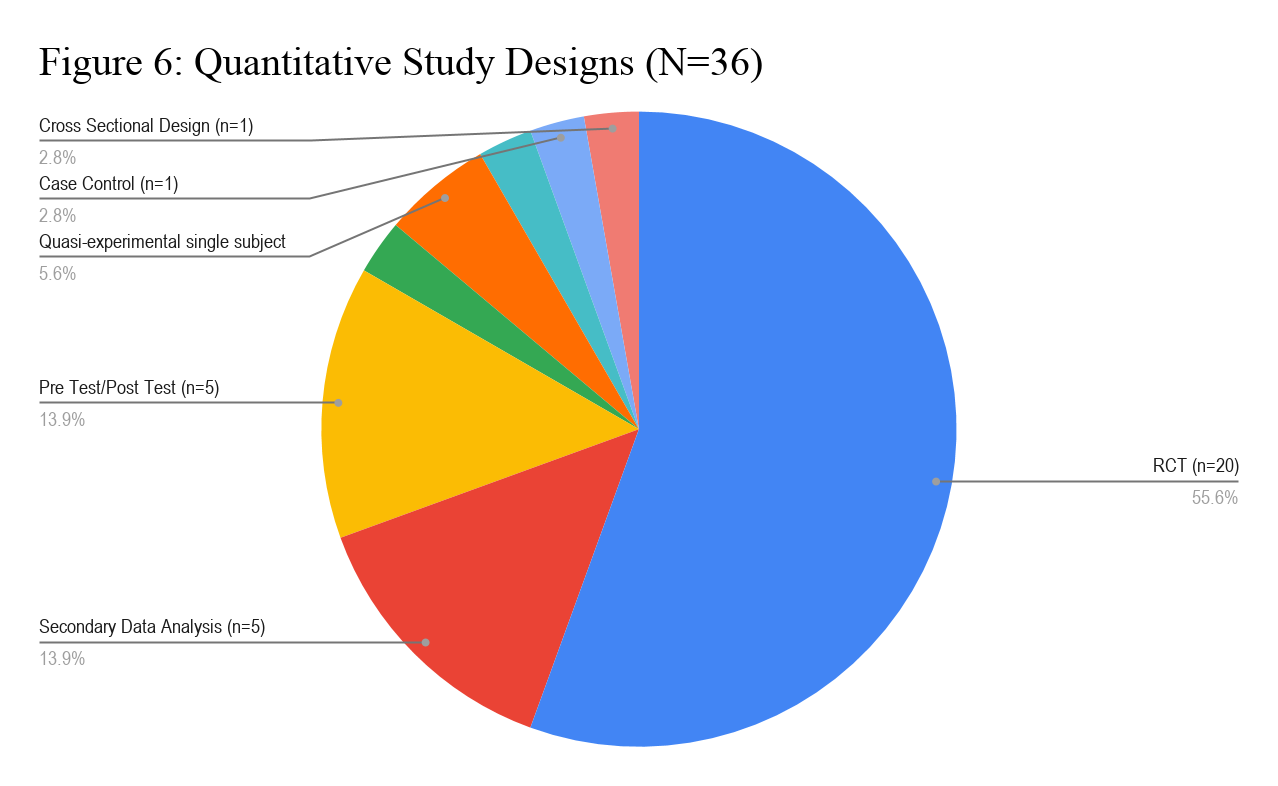


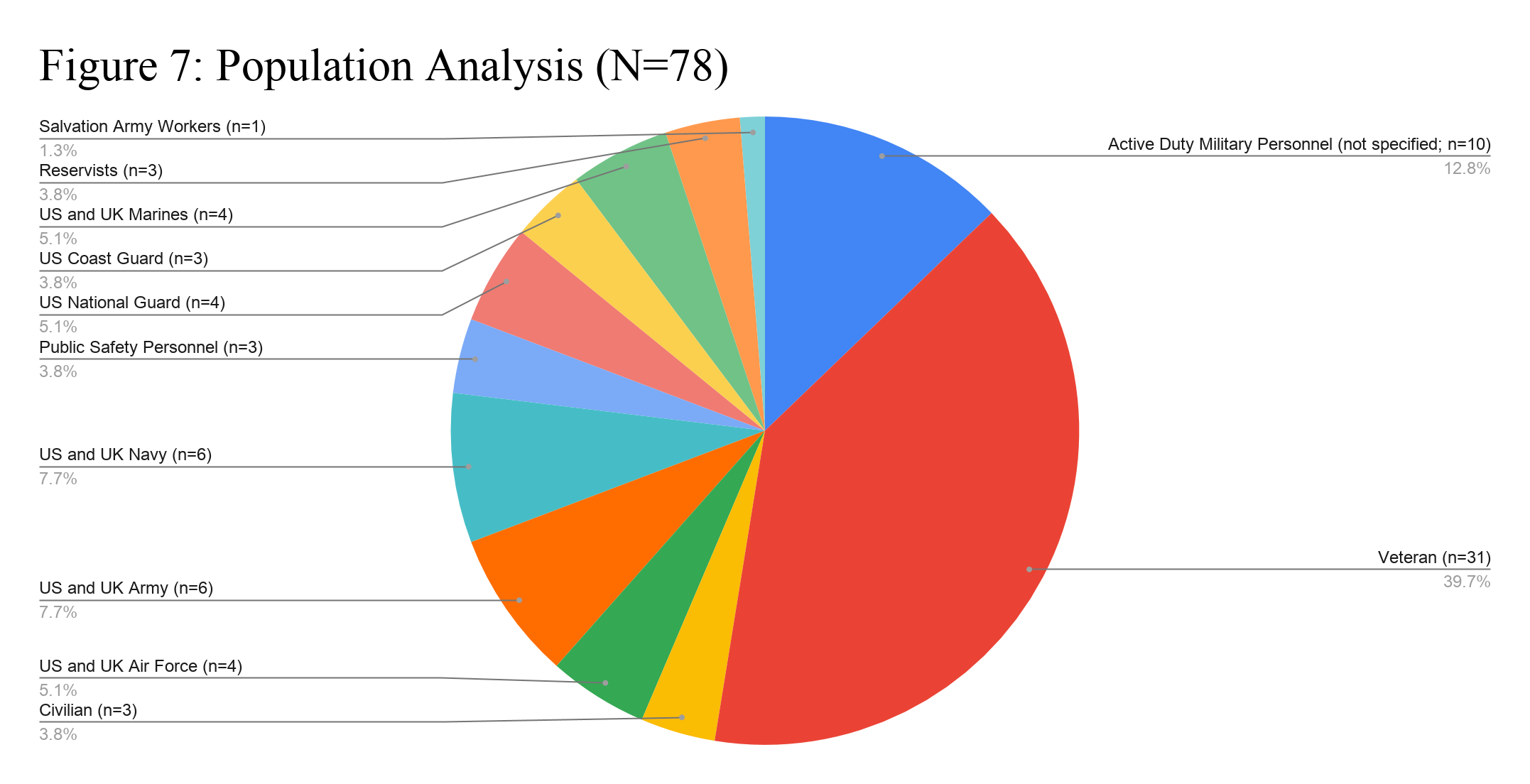


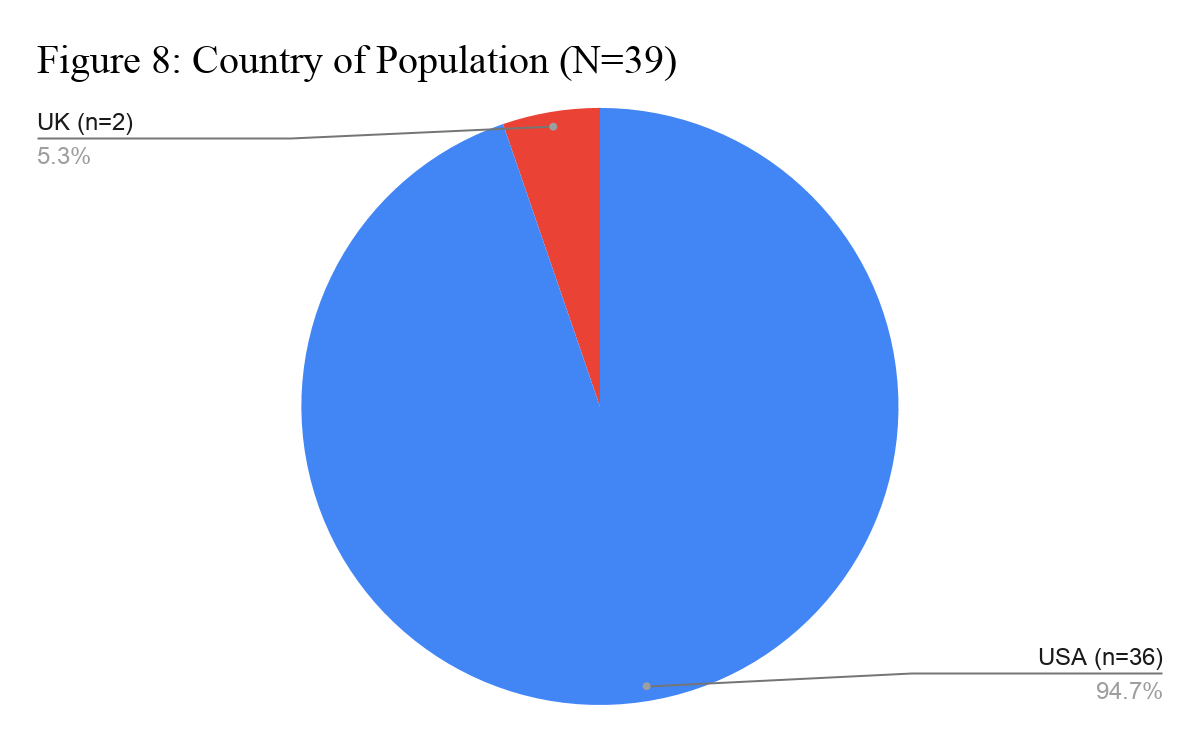


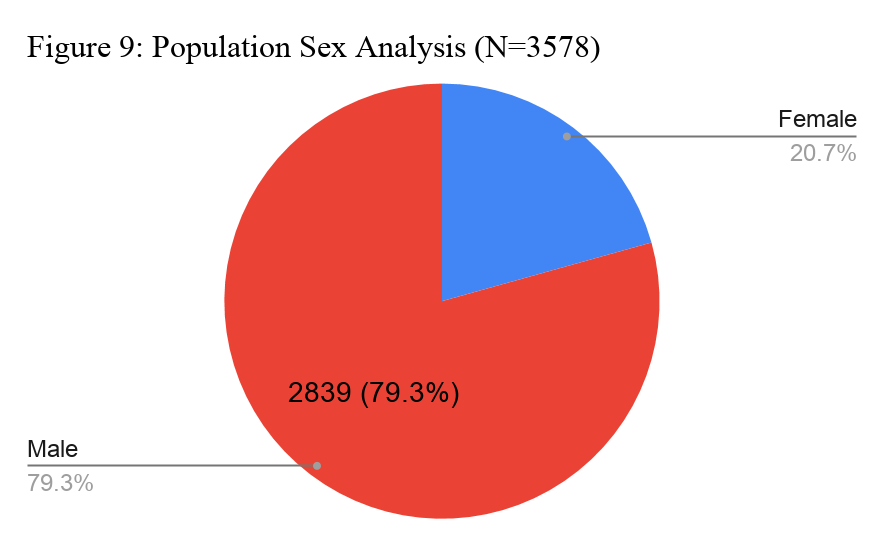

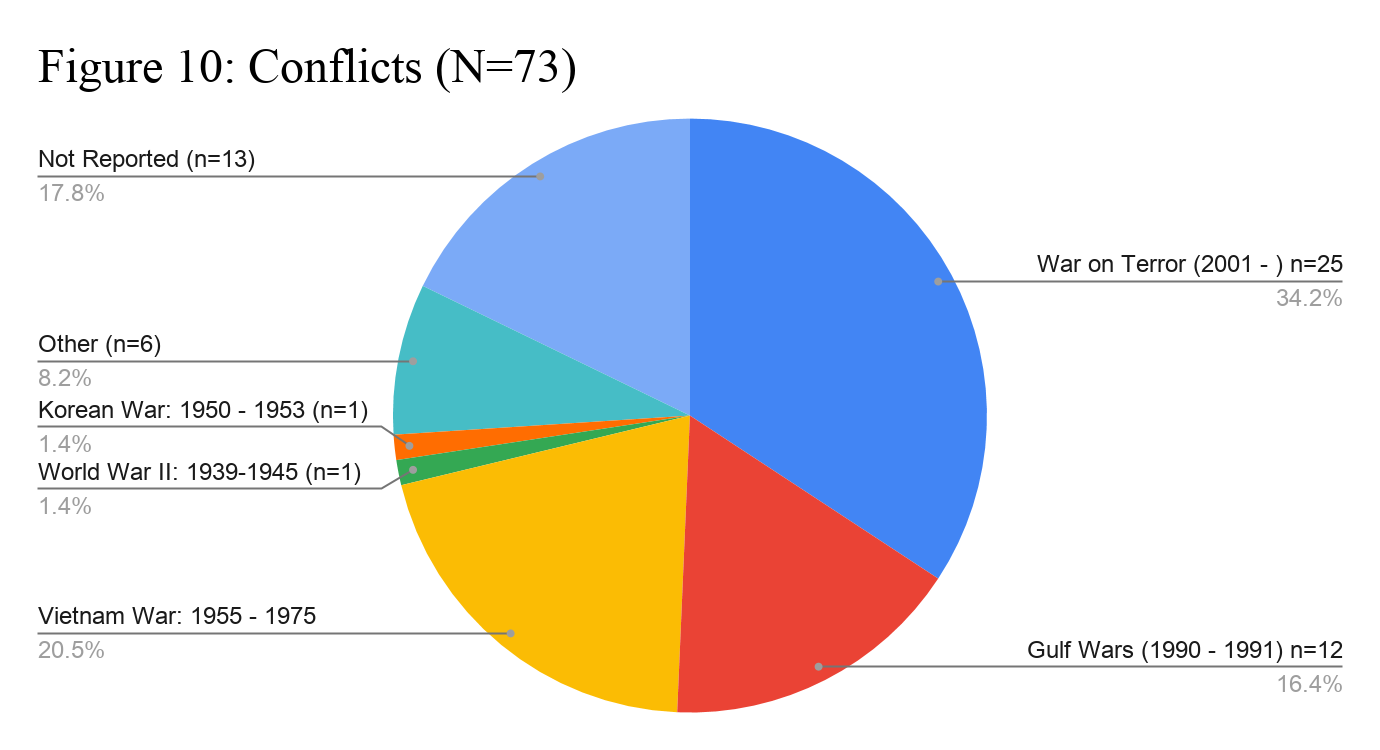

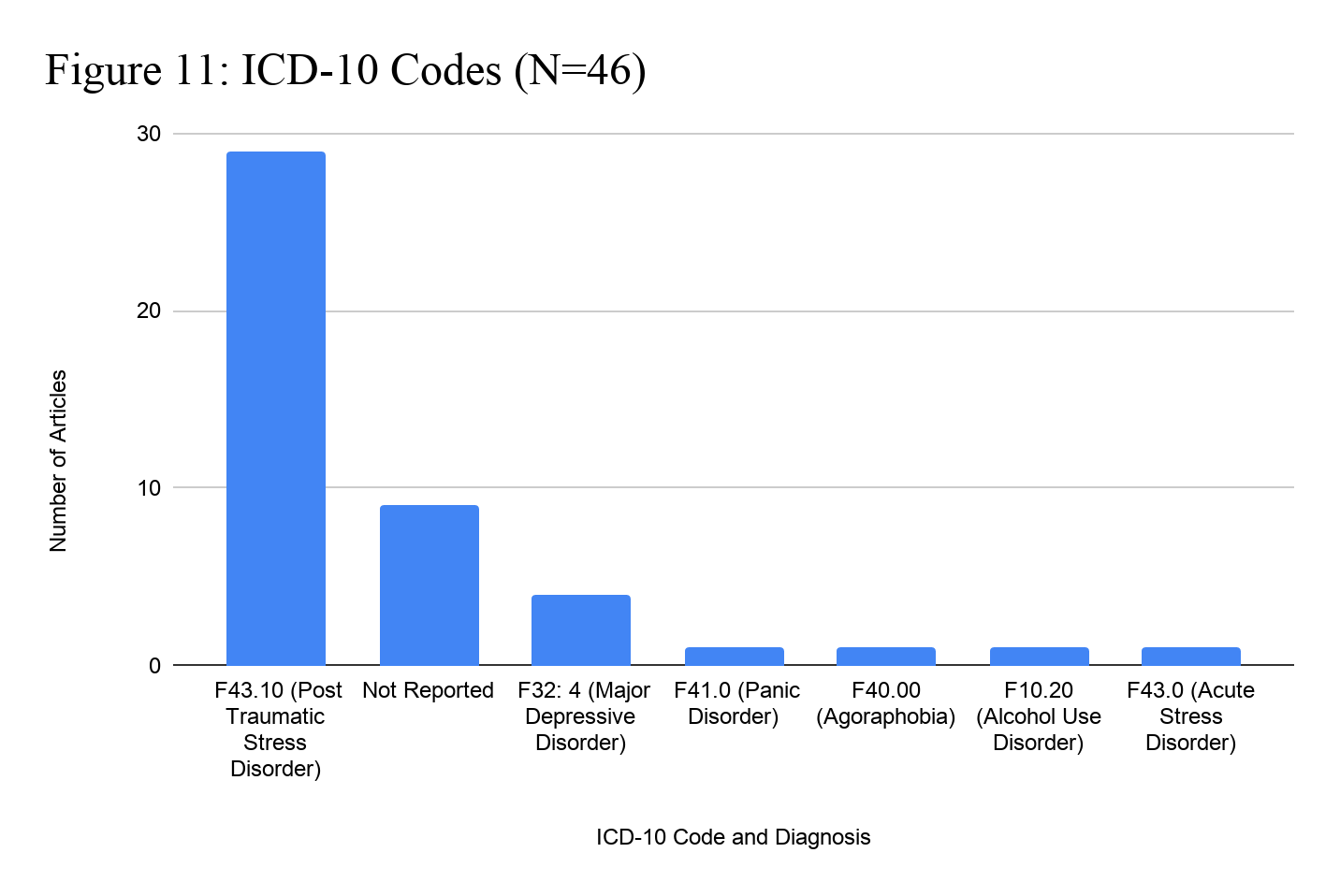

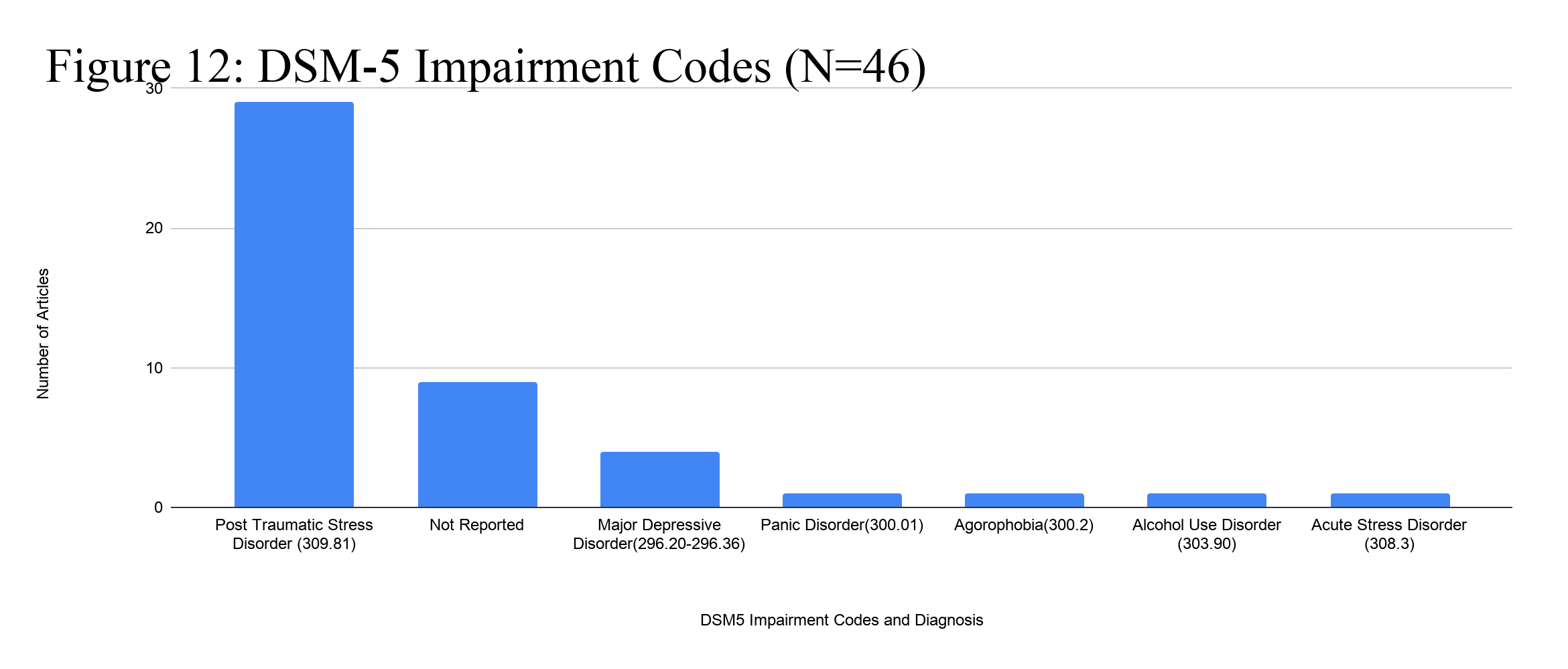


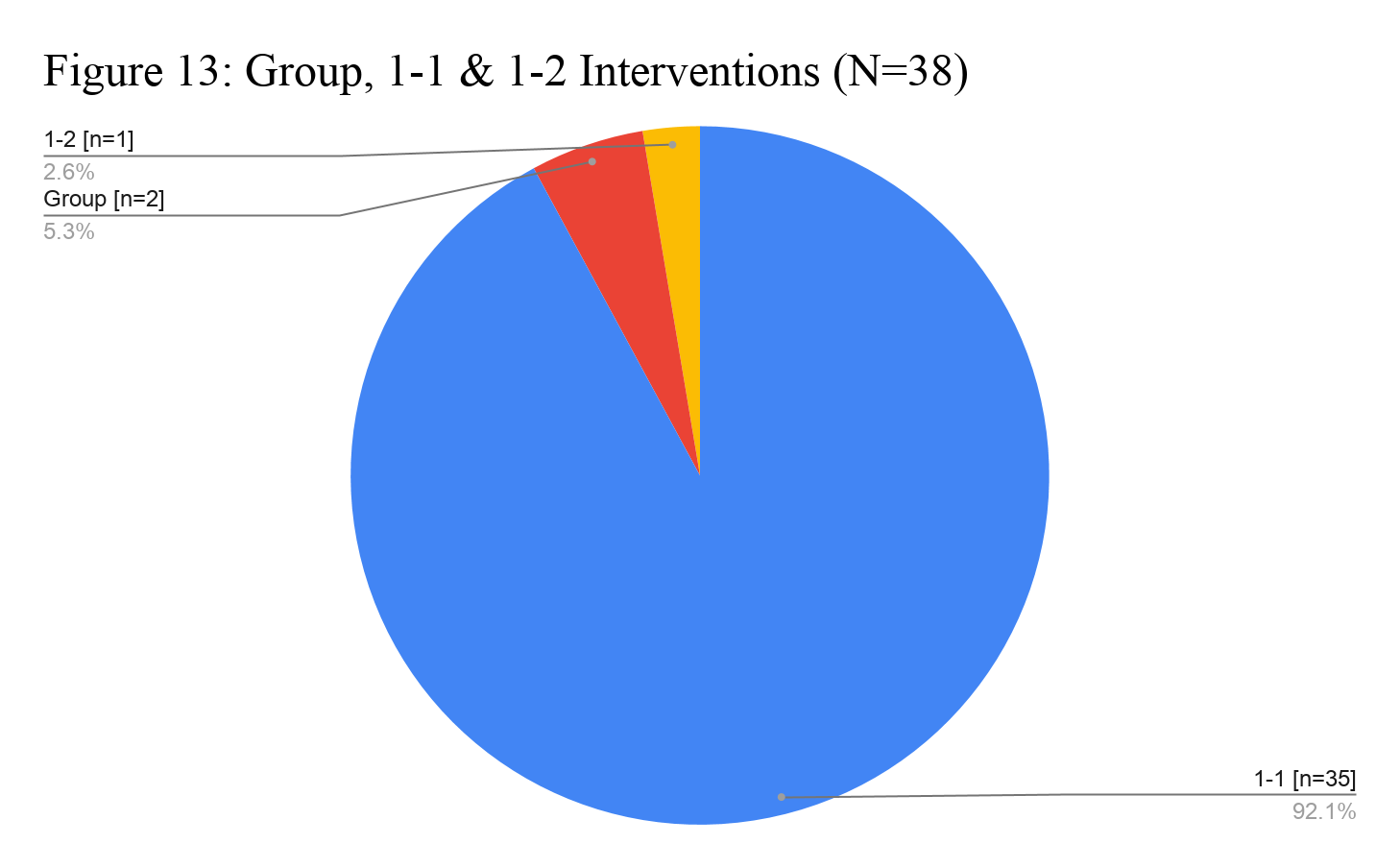

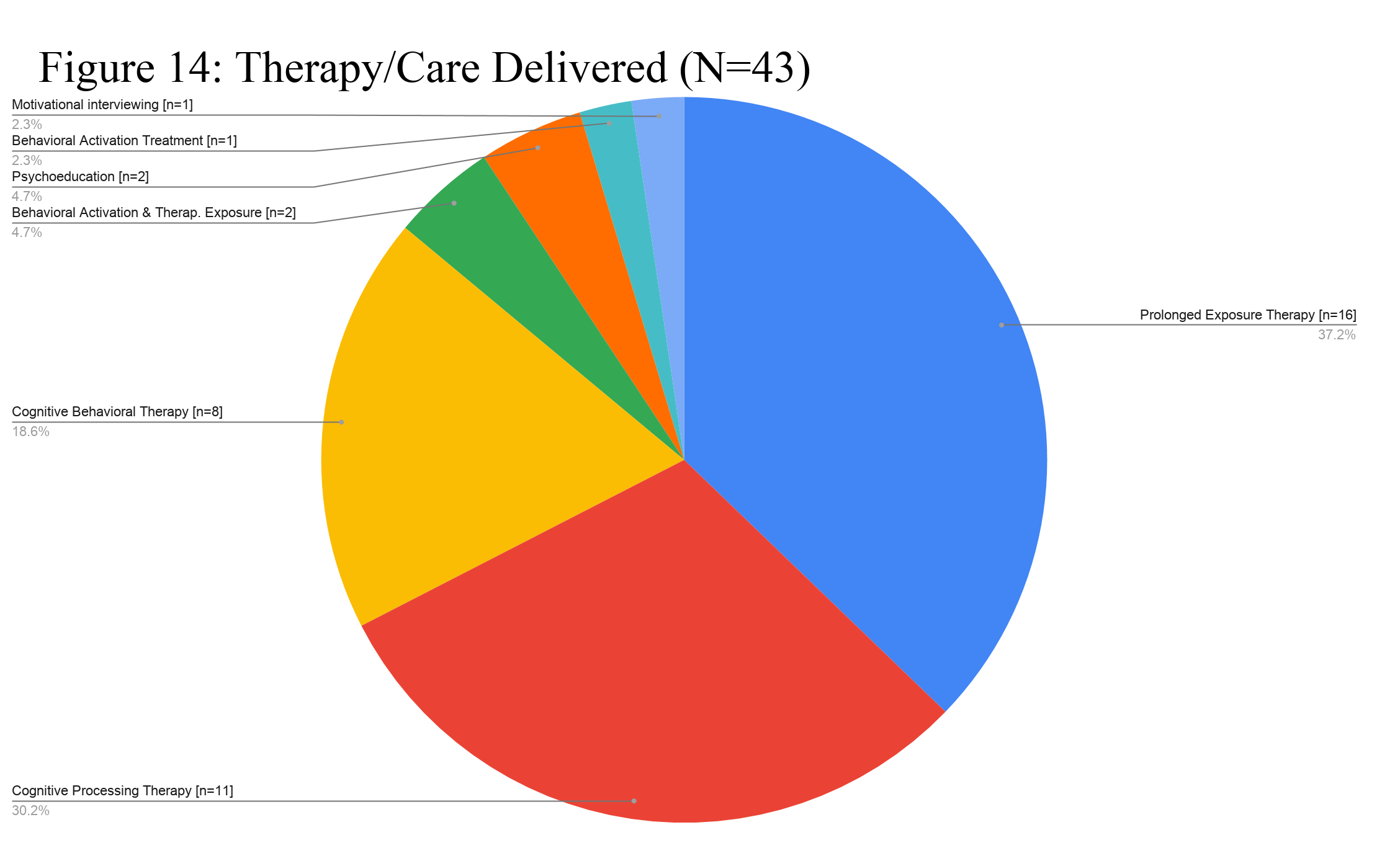

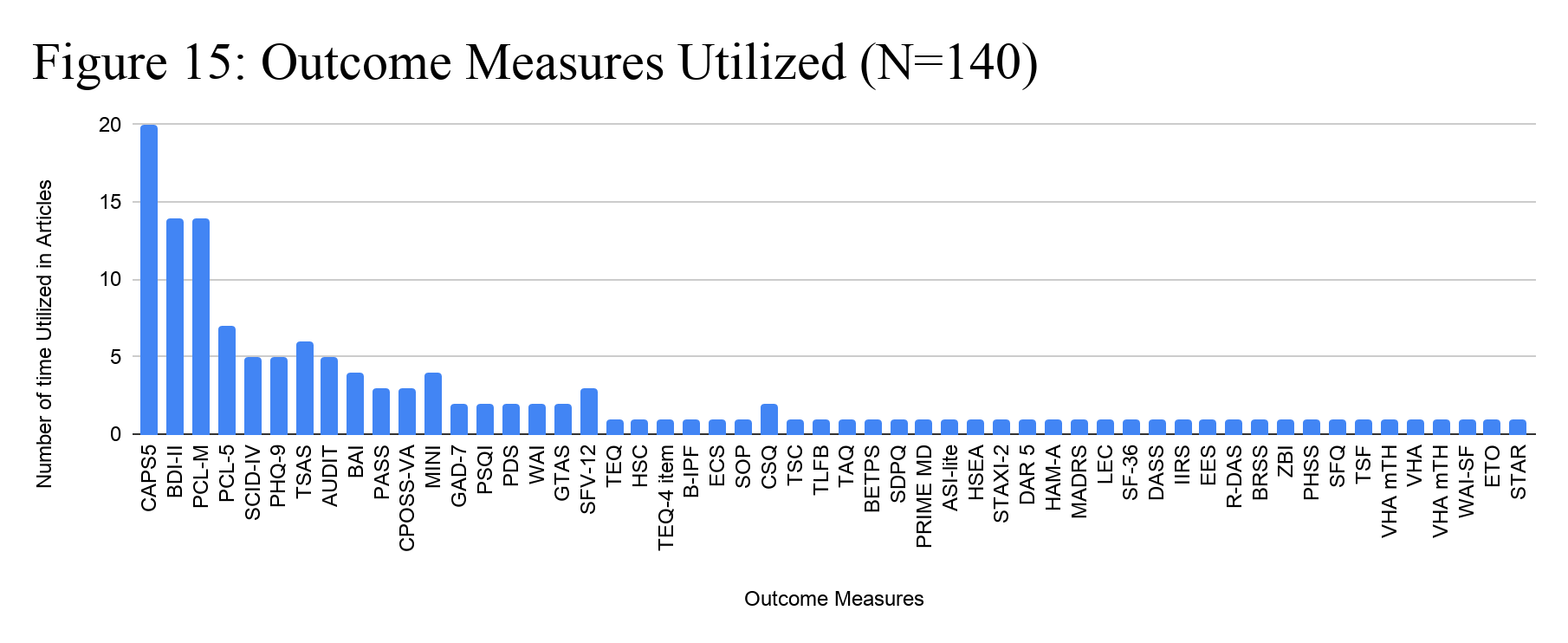


CAPS5 (Clinician Administered PTSD Scale); Beck’s Depression Inventory (BDI-II); PTSD Checklist for Military (PCL-M); PTSD Checklist DSM5 (PCL-5); Structured Clinical Interview for the DSM-IV (SCID-IV); Telemedicine Satisfaction and Acceptance Scale (TSAS); Alcohol Use Disorder Identification Test (AUDIT); Beck’s Anxiety Inventory (BAI); Perceptions about Services Scale (PASS); Charleston Psychiatric Outpatient Satisfaction Scale-VA version (CPOSS-VA); Mini-International Neuropsychiatric Interview (MINI); Generalized Anxiety Disorder 7 item Scale (GAD-7); Pittsburgh Sleep Quality Index (PSQI); Posttraumatic Diagnostic Scale (PDS); Working Alliance Inventory (WAI); Group Therapy Alliance Scale (GTAS); Veterans' SF-12 Health Survey (SFV-12); Treatment Expectancy Questionnaire (TEQ); Hopkins Symptom Checklist (HSC); 4-Item Treatment Expectancy Questionnaire (TEQ-4); Brief Inventory of Psychosocial Functioning (B-IPF); Exercise Confidence Survey (ECS); Suicide Assessment and Risk Management Standard Operation Procedure (SOP); Client Satisfaction Questionnaire (CSQ); Treatment Session Checklist (TSC); Timeline Followback (TLFB); Telehealth Attitudes Questionnaire (TAQ); Barriers to Exposure Therapy Participation Scale (BETPS); Service Delivery Perceptions Questionnaire (SDPQ); Primary Care Evaluation of Mental Disorders (PRIME MD); Addiction Severity Index Lite (ASI-lite); HELP Self-Efficacy Assessment (HSEA); State-trait Anger Expression Inventory 2 (STAXI-2); Dimensions of Anger Reactions (DAR 5); Hamilton Anxiety Rating Scale (HAM-A);Montgomery-Asberg Depression Rating Scale (MADRS); Life Events Checklist (LEC); Rand 36 item Short Form Health Survey (SF-36); Depression Anxiety and Stress Scale (DASS); Illness Intrusiveness Ratings Scale (IIRS); Eating Efficacy Scale (EES); The Revised Dyadic Adjustment Scale (R-DAS); Burns Relationship Satisfaction Scale (BRSS); Zarit Burden Interview-short version (ZBI); Patient Health Satisfaction Survey (PHSS); Sexual Functioning Questionnaire (SFQ); Treatment Satisfaction Form (TSF); VHA mTH Patient Satisfaction Questionnaire (VHA mTH PSQ); VHA Mobile Telehealth Questionnaire (VHA mTH); VHA Perception of HTMH Intervention Questionnaire, Working Alliance Inventory Short Form (WAI-SF); Expectancy of Treatment Outcome (ETO); STAR Scale to Access Therapeutic Relationships in Community Mental Health Care (STAR Scale)


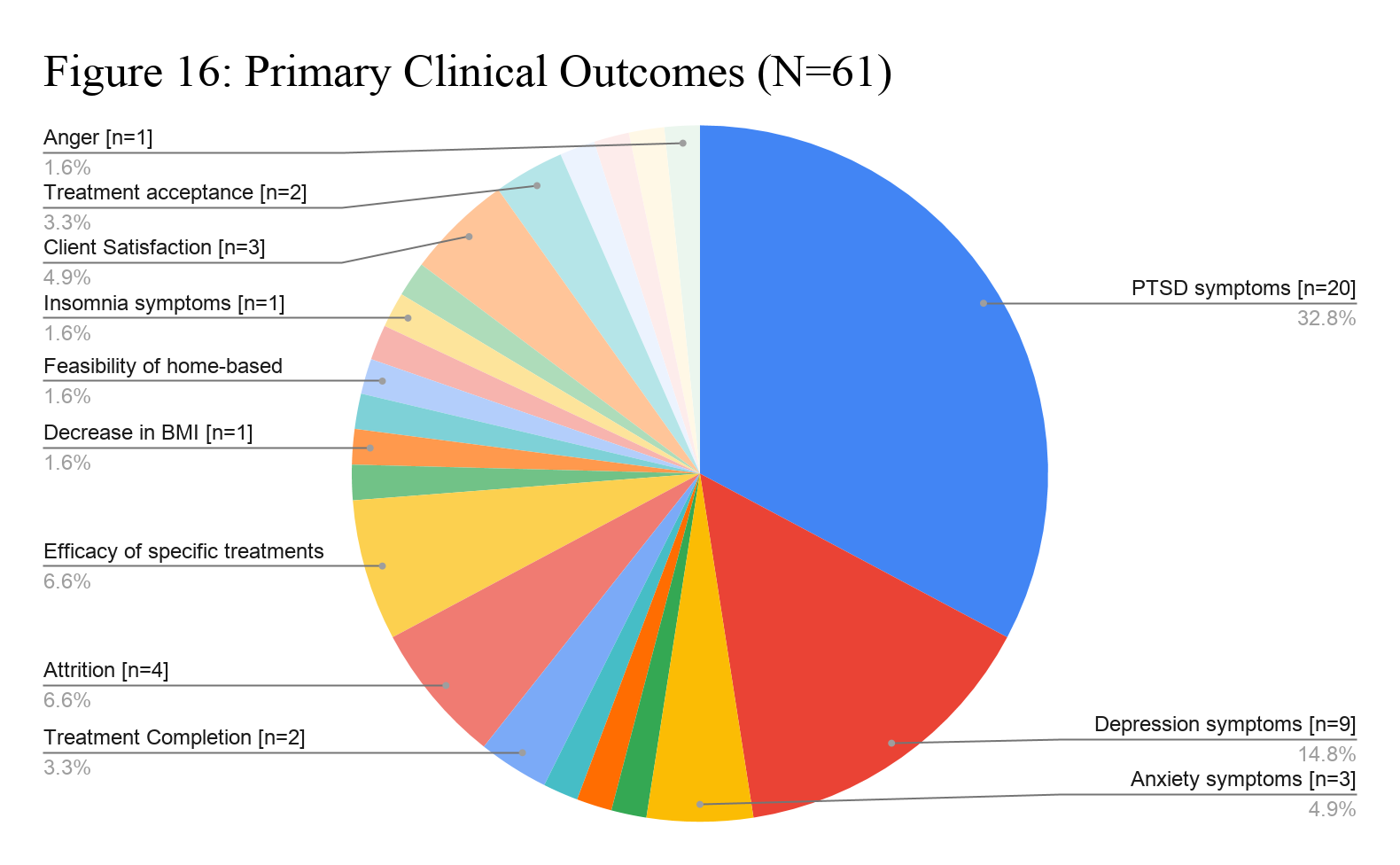


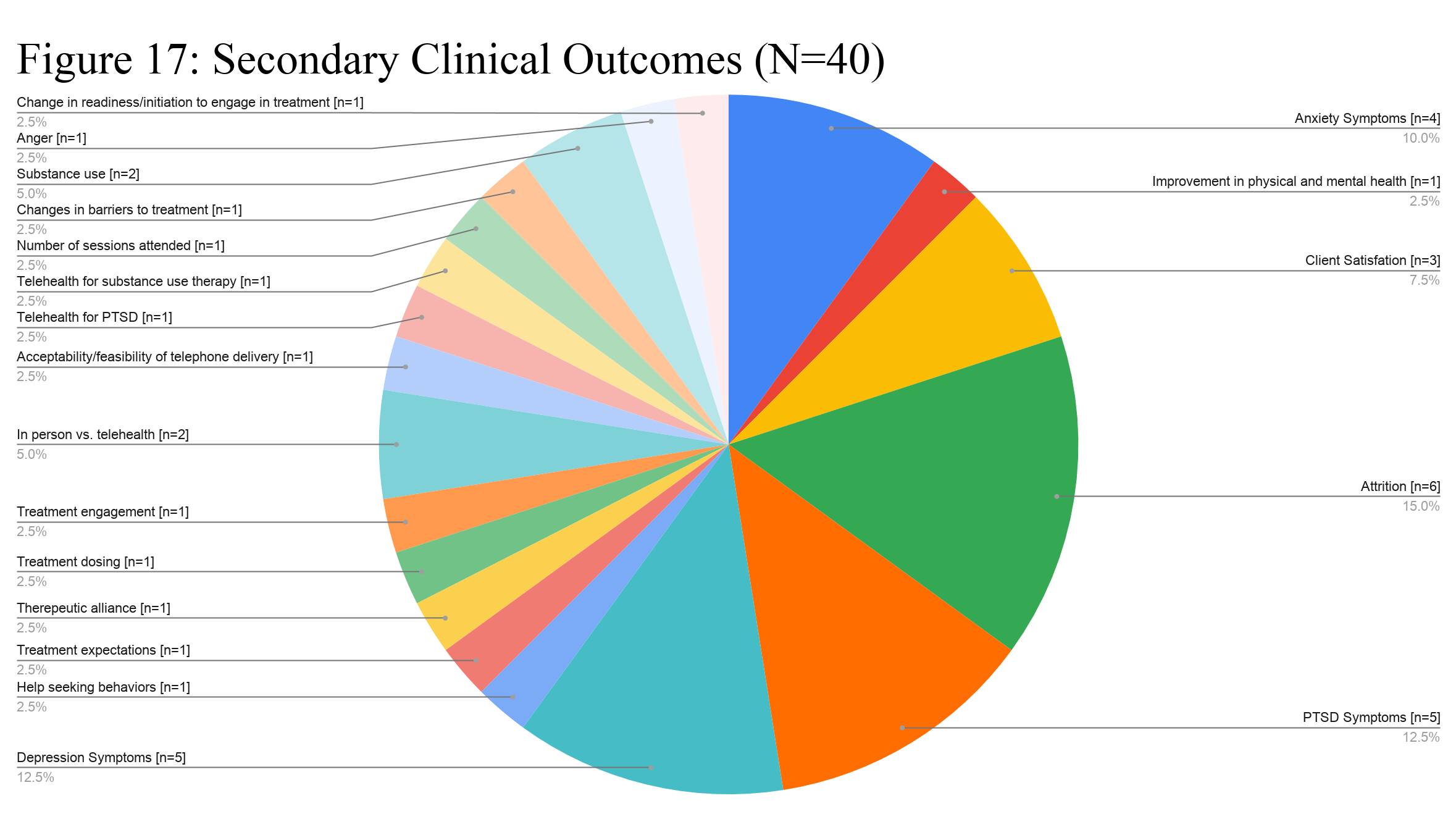

Supplement: Multimedia Appendix 2 [file mhealth_v8i9e22079_app2.docx]
