# Supplementary material for: Virtual Trauma-Focused Therapy for Military Members, Veterans, and Public Safety Personnel With Posttraumatic Stress Injury: Systematic Scoping Review
Source: JMIR Mhealth Uhealth. 2020 Sep 21;8(9):e22079. doi: 10.2196/22079 (PMC7536597; doi:10.2196/22079)
Supplement: Multimedia Appendix 3 [file mhealth_v8i9e22079_app3.docx]

| Study | Study design | Country | Population | Sex (%) | Race (%) | Therapy delivered | Mode of delivery | PEDro^a^ score | Outcome of interest (scales) and effectiveness |
| --- | --- | --- | --- | --- | --- | --- | --- | --- | --- |
| Ziemba et al, 2014 | Quantitative, RCT^b^ | United States | Active duty militaries and veterans | Male (90), female (10) | Black (79), other (21) | CBT^c^ | Telehealth (unspecified) | 6 | Significant PTSD^d^ symptom decrease pre and post-DH^e^ CBT (CAPS-5^f^, HAM-A^g^, and MADRS^h^; *P*=NR^i^); nonsignificant difference in effectiveness between CBT delivered in-person and via telehealth (*P*=NR); both in-person and DH CBT modes of delivery were effective |
| Yuen et al, 2015 | Quantitative, RCT | United States | Veterans | Male (98.1), female (1.9) | White (53.8), Black (36.5), Hispanic (9.6) | PE^j^ | Videoconference | 9 | Significant PTSD, depression, and anxiety symptom decrease pre/post DH PE (CAPS-5, PCL-M^k^, BDI-II^l^, and BAI^m^; *P*<.001); DH PE appeared to be effective for MH^n^ symptom reduction |
| Wierwille et al, 2016 | Quantitative, secondary data analysis | United States | Veterans | Male (87.8), female (12.2) | Minority status (14.4), other (85.6) | PE, CPT^o^ | Videoconference | N/A^p^ | Clinically significant PTSD and depressive symptom decreased pre/post DH CPT/PE (PCL-5, *P*<.001; BDI-II, *P*=.007); nonsignificant difference between CBT delivered in-person and via telehealth (*P=*.32) |
| Whealin et al, 2017 | Quantitative, survey based pretest-posttest | United States | Veterans and civilians (family members) | Veterans: male (96), female (4);  civilians: female (96), male (4) | Veterans: NH/PI^q^ (60.7), Asian American (10.7), White (21.4%), Black (3.6), NR (3.6); civilians: NH/PI (53.6), Asian American (21.4), White (17.9), Black/African American (3.57), NR (3.57) | CBT, psychoeducation | Videoconference | N/A | Overall relationship quality scores for veterans were significantly higher pre/post DH CBT/psychoeducation (RDAS^r^, *P*=.008); relationship satisfaction not significantly changed pre/post (BRSS^s^, *P*=.063); the intervention was effective at improving scores on measures of relationship quality, satisfaction, and cohesion pre/post for veterans and their family members |
| Whealin et al, 2017 | Quantitative, survey based | United States | Veterans | Male (83), female (17) | NH/PI (27.6), White (25.6), Asian American (21.3), Mixed (19.1), Black (6.4) | CPT | Videoconference | N/A | The intervention was well received by a rural, largely minority group of veterans as a means of obtaining MH care in a timely fashion (VHA^t^ mTH^u^, VHA perceptions of HTMH^v^ intervention questionnaire, and VHA mTH PSQ^w^; *P=*NR) |
| Wells et al, 2019 | Quantitative, secondary data analysis | United States | Civilians and veterans | Female (100%) | NR | CPT | Videoconference | N/A | Statistically significant increases in arousal, satisfaction, and desire pre/post DH CPT; the larger the reduction in post-PTSD symptoms, the larger the improvement in sexual function (CAPS-5 and SFQ^x^; *P>.*05); DH CPT is as effective as in-person delivery |
| Valentine et al, 2019 | Quantitative, secondary data analysis | United States | Veterans | Female (73.5), male (26.5) | White (68.5), African American (22.8), Latinx (3.5) | CPT and PE | Videoconference | N/A | Survivors of MST^y^ were significantly more likely to leave DH intervention earlier and not complete treatment than in-person therapy group (MINI^z^ and CAPS, *P*=.04 and *P*=.04); survivors of MST were less likely to receive a minimum adequate dose of trauma-focused treatment and that early attrition was particularly salient when care was delivered remotely via DH |
| Tuerk et al, 2010 | Quantitative, case control | United States | Combat veterans | Male (97), female (3) | Black (34), White (64), Hispanic (2) | PE | Videoconference | N/A | Significant PTSD and depression symptoms decreased pre/post DH PE (PCL-M and BDI-II; *P*>.001); nonsignificant difference in effectiveness between PE delivered in-person and via telehealth (*P*>.001); both in-person and DH PE modes of delivery were effective |
| Trahan et al, 2016 | Quantitative, quasi-experimental single-subject design | United States | Veterans | Female (100%) | African American | CBT | Telephone | N/A | The participant’s PTSD, anxiety, and depression symptoms were reduced postintervention and quality of life scores increased (PHQ-9^a,a^, PCL-5^a,b^, GAD-7^a,c^, and PCS^a,d^) |
| Strachan et al, 2012 | Quantitative, RCT | United States | Veterans | Male (92.5), female (7.5) | White (45), other (55%) | Behavioral activation and therapeutic exposure | Videoconference | 7 | Reductions in PTSD symptoms were significant for both DH and in-person therapy while changes in depressive symptoms were not (CAPS^a,e^, PCL-M, SCID-IV^a,f^, BDI-II, BAI, and AUDIT^a,g^; *P*=.009); home-based DH application of behavioral health treatments was a feasible treatment delivery method |
| Stecker et al, 2013 | Qualitative, exploratory | United States | Active duty, National Guard and reservists, and separated | Male (84), female (16) | White (67), African American (13), Latino (9) | CBT | Telephone | N/A | Four categories of beliefs were associated with the decision to seek treatment, including concerns about treatment, emotional readiness for treatment, stigma, and logistical issues and suggests areas for intervention efforts to minimize barriers to treatment |
| Stecker et al, 2014 | Quantitative, RCT | United States | Service members | Male (87), female (13) | White (69), African American (14), other (14) | CBT | Telephone | 5 | Those who received the CBT telephone intervention attended a significantly greater number of treatment sessions in the following 6 months than the control group participants (PCL-M, PHQ-9, and PASS^a,h^; *P*=.04); a one-time brief telephone intervention could engage service members in PTSD treatment earlier than conventional methods and could lead to immediate symptom reduction |
| Stecker et al, 2016 | Mixed methods, RCT and general qualitative exploratory | United States | Service members | Male (87), female (13) | White (83.4), Black (16.6) | CBT | Telephone | 4 | Black participants were significantly more likely to initiate DH CBT treatment (*P*=.04) compared with White participants, although they attended fewer overall sessions (MINI, PCL-M, and PASS); all participants had a significant decrease in PTSD (*P*=.0001) and depression (*P*=.001) symptoms; 3 themes of PTSD, treatment-seeking behavior, and efforts toward symptom reduction emerged: (1) social connectedness, (2) attitudes and expectations toward treatment, and (3) the desire to appear *ok* |
| Morland et al, 2015 | Quantitative, RCT | United States | Civilians and veterans | Female (100%) | White (47.6), Asian (14.3), Pacific Islander (11.9), other (26.2) | CPT | Videoconference | 5 | DH CBT outcomes were comparable to in-person treatment reducing PTSD symptoms when compared with the civilian population (CAPS, TEQ^a,i^, WAI^a,j^, and CPOSS-VA^a,k^; *P*=.001); results indicated acceptability and safety of telemedicine for women with PTSD |
| Morland et al, 2014 | Quantitative, RCT | United States | Combat veterans | Male (100%) | Asian (15.2), White (46.4), Pacific Islander (13.6), other (Hispanic, Black, and Native American; 16) | CPT-cognitive only | Videoconference | 8 | No significant difference in effectiveness of CPT-cognitive only delivered in-person and via DH (CAPS, CPOSS-VA, TSAS^a,l^, TEQ 4-Item^a,m^, and GTAS^a,n^; *P*=.24); videoconferencing was found to be comparable with in-person treatment; all participants saw a reduction in PTSD symptoms posttreatment and at follow-up |
| Morland et al, 2019 | Quantitative, RCT | United States | Veterans, Air Force, Army, Navy, Coast Guard, and National Guard | Male (100%) | American Indian/American Native (2.9), Black (28.6), White (40.6), Asian American (8.0), Native Hawaiians or Other Pacific Islander (2.3), other (9.7) | PE | Telehealth (unspecified) | 7 | No significant difference in effectiveness between in-person or DH delivery of PE (CAPS5, BDI-II, B-IPF^a,o^, and AUDIT; *P*>.385); clinical effectiveness of prolonged exposure did not differ between home-based telehealth, office-based telehealth, or in-home-in-person treatment; however, completion of treatment was higher in in-home-in-person treatment in comparison with home (*P*=.03) or office-based (*P*<.001) telehealth |
| Morland et al, 2011 | Quantitative, RCT | United States | Active duty reserves, guard, and veterans; army, marines | Male (100%) | White (31), NH/PI (46.1), African American (15.4), Asian (7.7) | CPT- cognitive only | Videoconference | 6 | No significant differences between in-person versus DH CPT-cognitive only on treatment dropout (*P*=.26), between conditions in the number of sessions attended, medians of total number of completed homework assignments, scores between treatment conditions on treatment expectancy measure, or treatment conditions at posttreatment and at 6-month follow-up (CAPS, GTAS, and TSAS; *P*>.05) |
| Maieritsch et al, 2016 | Quantitative, RCT | United States | Veterans | Male (93), female (7) | NR | CPT | Videoconference | 5 | All participants receiving in-person and DH CPT experienced a reduction of PTSD (CAPS, *P*=.09 and PCL-5, *P*=.08) at posttreatment assessment; no significant differences were observed between in-person and DH CPT interventions (CAPS, SCID-I SCID-IV, PCL-5, BDI-II, and WAI) |
| Luxton et al, 2015 | Quantitative, pretest-posttest | United States | Active duty military | Male (100%) | NR | Behavioral activation treatment | Videoconference | N/A | Statistically significant reduction in PTSD symptoms and severity (CAPS, PCL-M, BDI-II, BAI, PSQI^a,p^, SOP^a,q^, CSQ^a,r^, and TSC^a,s^); feasibility of home-based therapy for active military personnel was found |
| Franklin et al, 2018 | Quantitative, RCT | United States | Veterans | Male (100%) | African American (66), European American (28), other (6) | CBT for insomnia | Telephone | 7 | No difference in mode of delivery (in-person or DH; *P*=.28), PSQI scores immediately posttreatment (*P*=.33), at 1 month (*P*=.21), or 3 months posttreatment; large effects for in-person CBT for insomnia at all 3 time points and medium-to-large effects for telephone-delivered CBT for insomnia (CAPS, SCID-IV, and PSQI); in-person worked faster; however, there was no difference between groups at the 3-month posttreatment mark |
| Jaconis et al, 2016 | Quantitative, quasi-experimental single-subject design | United States | Veterans | Female (100%) | Black | PE | Videoconference | N/A | Participants were able to complete 12 sessions of therapy and experienced clinically significant reductions in PTSD, depression symptoms, and a reduction in alcohol use, all of which were maintained at 3 and 6 months follow-up (BDI-II, CAPS, PCL-M, and TLFB^a,t^) |
| Hernandez-Tejada et al, 2014 | Quantitative, RCT | United States | Military personnel | Male (100%) | White (57.8), other (42.2) | PE | Telehealth (unspecified) | 4 | DH participants and in-person participant dropout rates were comparable; DH did not predict lower dropout rates (BETPS^a,u^, TAQ^a,v^, PCL-M, and BDI-II) |
| Hernandez-Tejada et al, 2017 | Quantitative, pretest-posttest | United States | Veterans | Male (69), female (31) | Black (69), White (31) | PE | Telephone | N/A | PHQ-9, PCL-M: peer support was a promising tool that could be used to combat dropout rates in PE for PTSD in military personnel and veterans regardless if delivered in-person or via DH (*P=.*03) |
| Grubbs et al, 2015 | Quantitative, secondary data analysis | United States | Veterans | Male (88.7), female (11.2) | White (57.9), African American (23.3), Hispanic (9), other (9.8) | CPT | Videoconference | N/A | Higher CAPS scores and the opt-out recruitment method predicted lower likelihood of initiating DH CPT; diagnosis of major depressive disorder lowered likelihood of engaging in CPT; a pending Veterans Affairs disability claim was a positive predictor for engagement in DH CPT (MINI, AUDIT, CAPS, and SFV-12^a,w^) |
| Gros et al, 2011 | Quantitative, pretest-posttest | United States | Veterans | Male (93.5), female (6.5) | White (50), African American (45.2), NR (4.8) | PE and psychoeducation | Videoconference | N/A | DH condition produced significant reduction in PTSD symptoms and depression, but was not as effective as the in-person condition (PCL-5, BDI-II, DASS^a,x^, and IIRSp^a,y^; *P*<.01); DH and in-person treatment were effective in treating PTSD and depression in military veterans |
| Gros et al, 2018 | Quantitative, RCT | United States | Veterans and military personnel | Male (94), female (6) | White (55.2), Black (38.8), other (6) | PE | Videoconference | 6 | PTSD symptoms were significantly decreased in both the DH and in-person conditions (CAPS, CPOSS^a,z^, and SDPQ^b,a^; *P*<.001); there were no significant effects of modality on any of the measures of the perception of the quality of service delivery and satisfaction with services provided (*P*<.16) |
| Gallegos et al, 2016 | Mixed methods, RCT and thematic analysis | United States | Veterans | Male (87.2), female (12.8) | White (70.6), Black (14.5), Hispanic (7.8), Asian American (1.9), American Indian or Alaskan Native (2.2), other (3.0) | CBT | Telephone | 6 | Significant reduction in PTSD and depression symptoms postintervention (*P*<.01); no significant treatment seeking (DH CBT) difference between suicide and nonsuicidal groups (PCL-M, PASS; *P<*.05) |
| Franklin et al, 2017 | Quantitative, RCT | United States | Veterans | Male (92.3), female (7.7) | Euro-American (75), African American (8.3), other (16.7) | PE | Videoconference | 5 | Those who had some form of PE had statistically significant PTSD symptom reduction (CAPS, *P=.*02; PDS, *P*=.01) compared with the treatment as usual group (CAPS, PDS^b,b^, BDI-II, and BAI); there was no significant difference in symptom reduction among those who attended PE via DH or in-person |
| Fortney et al, 2015 | Quantitative, RCT | United States | Veterans | Male (89.8), female (10.2) | White (63.8), African American (9.6), Hispanic (7.6), other (9) | CPT | Telephone | 7 | Telemedicine outreach for PTSD increased DH CPT initiation and engagement in therapy compared with the control group (*P<*.001), which contributed to statistically significant PTSD symptom reductions at 6 (*P*<.001) and 12 months (*P*=.02) follow-up for patients (PDS; MINI, AUDIT, and SFV-12) |
| Seal et al, 2012 | Quantitative, RCT | United States | Veterans | Male (64), female (36) | Caucasian/White (45.2), Black/African American (8.2), Asian (19.2), Hispanic/Latino (19.2), multiracial/other (8.2) | Motivational interviewing | Telephone | 7 | A greater proportion of those randomized to the DH motivational interviewing versus control group engaged in MH treatment (PCL-M, PHQ-9, PRIME MD^b,c^, AUDIT, and ASI-lite^b,d^; *P*=.004); those engaged in motivational interviewing significantly decreased marijuana use (*P*<.05) and MH treatment–related stigma (*P*=.03) and increased self-reported intention to engage in MH treatment (8 weeks, *P*=.02; 16 weeks, *P*=.005) |
| Pelton et al, 2015 | Quantitative, single case design | United States | Military | Male (100%) | NR | PE | Videoconference | N/A | Continued decline in symptoms after switching from in-person to DH PE sessions during the second half of the treatment (PCL-M); study supports the delivery of PE therapy via DH without a discernible negative impact or compromise to the therapeutic alliance |
| Olden et al, 2017 | Quantitative, RCT | United States | Military, public safety personnel (Navy, Army reserves, Coast Guard, Army, firefighter, National Guard, law enforcement, Salvation Army workers) | Male (81.8), female (18.2) | White (63.7), Black (9.1), Hispanic (18.2), other (9.1) | PE | Videoconference | 4 | Improvement in PTSD and depressive symptoms (CAPS, *P*<.001; BDI-II, *P*=.004); half of study completers no longer met the criteria for PTSD and none met the criteria for major depression at posttreatment assessment; significant improvement in anger expression (STAXI-2^b,e^; *P*=.048; *d*=0.71); CAPS, BDI-II, PCL, STAXI-2, WAI-SF^b,f^, and CSQ^b,g^: individuals in occupations at risk with PTSD endorsed high levels of therapeutic alliance, treatment satisfaction, satisfaction with the use of videoconferencing, and satisfaction with clinical interaction |
| Murphy and Turgoose, 2019 | Quantitative, pretest-posttest | United Kingdom | Veterans | Male (88.9), female (11.1) | NR | CPT | Videoconference | N/A | Age, education, employment, and relationship status were related to therapy completion (PCL-5, PHQ-9, GAD-7, DAR 5^b,h^, and AUDIT); improvements in MH scores following DH CPT were observed with a large effect size and maintained at 3 months |
| Morland et al, 2019 | Quantitative, cross-sectional design | United States | Veterans (Army, Marines, Navy, Airforce, and Coast Guard) | Male (75), female (25) | White (46), African American (28), Asian American (9), American Indian or Alaskan Native (3), NH/PI (3), other (11) | PE | Videoconference | N/A | No specific therapeutic treatment was preferred over others; differences between preference for DH versus in-person was not statistically significant (*P*>.199); veterans seeking care for PTSD could benefit from having DH and in-person options |
| Boykin et al, 2019 | Quantitative, secondary data analysis | United States | Veterans | Male (66.1), female (33.8) | White (51.4), African American (45.9), other/multiracial (1.4) | CPT and PE | Videoconference | N/A | No demographic characteristics significantly predicted the completion of therapy when using DH; therapy type was a significant predictor of completion. Findings support greater emphasis on clinical expertise and competence in delivering therapies via DH rather than emphasizing predetermined patient criteria to identify which patients might benefit from DH |
| Ashwick et al, 2019 | Qualitative, general qualitative | United Kingdom | Veterans (Army, Navy, Royal Air Force, and Marines) | Male (94), female (6) | White (94), Black (6) | CPT | Videoconference | N/A | 5 key themes: (1) effect of your own environment, (2) importance of good therapeutic alliance, (3) technicalities and practicalities, (4) personal accountability, and (5) measuring change; overall, the veterans appeared to support the use of DH; veterans felt comfortable in their own homes, were able to establish a good rapport with therapists, and reported symptom improvements |
| Acierno et al, 2017 | Quantitative, RCT | United States | Veterans | Male (96.2), female (3.8) | White (60.6), Black (33.3), Hispanic (5.3), other (0.8) | PE | Videoconference | 8 | PE delivered via DH or in-person were effective at reducing PTSD (PCL-M, *P*<.0001) and depression symptoms (BDI-II in-person, *P*<.0001, vs BDI-II DH, *P*<.004); treatment gains following DH PE were largely maintained in this study (CAPS, BDI-II, and PCL-M) |
| Acierno et al, 2016 | Quantitative, RCT | United States | Veterans | Male (94.4), female (5.6) | White (50.4), Black (47.4), Hispanic (0.9), other (1.3) | Behavioral activation and therapeutic exposure | Videoconference | 6 | Behavioral activation and therapeutic exposure delivered via DH was as effective as behavioral activation and therapeutic exposure delivered in-person in terms of its impact on reducing PTSD and major depressive disorder symptoms (CAPS, PCL-M, and BDI-II) |

^a^PEDro: Physiotherapy Evidence Database score.

^b^RCT: randomized controlled trial.

^c^CBT: cognitive behavioral therapy.

^d^PTSD: posttraumatic stress disorder.

^e^DH: digital health.

^f^CAPS-5: Clinician-Administered posttraumatic stress disorder Scale for Diagnostic and Statistical Manual of Mental Disorders, 5th edition.

^g^HAM-A: Hamilton Rating Scale for Anxiety.

^h^MADRS: Montgomery-Åsberg Depression Rating scale.

^i^NR:

^j^PE: prolonged exposure therapy.

^k^PCL-M: posttraumatic stress disorder checklist–military version.

^l^BDI-II: Beck Depression Inventory-II.

^m^BAI: Beck Anxiety Inventory.

^n^MH: mental health.

^o^CPT: cognitive processing therapy.

^p^N/A: not applicable.

^q^NH/PI: Native Hawaiian/Pacific Islander.

^r^RDAS: Revised Dyadic Adjustment Scale.

^s^BRSS: Burns Relationship Satisfaction Scale.

^t^VHA:

^u^mTH:

^v^HTMH: home telemental health.

^w^PSQ:

^x^SFQ: Sexual Function Questionnaire.

^y^MST: military sexual trauma.

^z^MINI: Mini-International Neuropsychiatric Interview.

^a,a^PHQ-9: Patient Health Questionnaire-9.

^a,b^PCL-5: PTSD checklist for DSM-5.

^a,c^GAD-7: General Anxiety Disorder-7.

^a,d^PCS: postconcussive symptoms.

^a,e^CAPS: Clinically Administered PTSD scale.

^a,f^SCID-IV: Structured Clinical Interview for DSM-IV.

^a,g^AUDIT: Alcohol Use Disorder Identification Test.

^a,h^PASS: perceptions about the services scale.

^a,i^TEQ: Treatment Expectancy Questionnaire.

^a,j^WAI: Working Alliance Inventory.

^a,k^CPOSS-VA: Psychiatric Outpatient Satisfaction Scale-VA version.

^a,l^TSAS: Telemedicine Satisfaction and Acceptance Scale.

^a,m^TEQ 4-Item: Treatment Expectancy Questionnaire 4-Item.

^a,n^GTAS: Group Therapy Alliance Scale.

^a,o^B-IPF: Brief Inventory of Psychosocial Functioning.

^a,p^PSQI: Pittsburgh Sleep Quality Index.

^a,q^SOP: Suicide Assessment and Risk Management Standard Operation Procedure.

^a,r^CSQ: Client Satisfaction Questionnaire.

^a,s^TSC: Treatment Session Checklist.

^a,t^TLFB: Timeline Followback.

^a,u^BETPS: Barriers to the Exposure Therapy Participation Scale.

^a,v^TAQ: Telehealth Attitudes Questionnaire.

^a,w^SFV-12: Veterans' SF-12 Health Survey.

^a,x^DASS: Depression Anxiety and Stress Scale.

^a,y^IIRS: Illness Intrusiveness Ratings Scale.

^a,z^CPOSS: Psychiatric Outpatient Satisfaction Scale.

^b,a^SDPQ: Service Delivery Perceptions Questionnaire.

^b,b^PDS: Posttraumatic Diagnostic Scale.

^b,c^PRIME MD: Primary Care Evaluation of Mental Disorders.

^b,d^ASI-lite: Addiction Severity Index.

^b,e^STAXI-2: State-trait Anger Expression Inventory 2.

^b,f^WAI-SF: Working Alliance Inventory Short Form.

^b,g^CSQ: Client Satisfaction Questionnaire.

^b,h^DAR 5: Dimensions of Anger Reactions.
